# Frailty in motion: Amnestic mild cognitive impairment and Alzheimer's disease cohorts display heterogeneity in multimorbidity classification and longitudinal transitions

**DOI:** 10.1177/13872877251319547

**Published:** 2025-03-02

**Authors:** Linzy Bohn, Yao Zheng, G Peggy McFall, Melissa K Andrew, Roger A Dixon

**Affiliations:** 1 Department of Psychology, University of Alberta, Edmonton, Alberta, Canada; 2 Neuroscience and Mental Health Institute, University of Alberta, Edmonton, Alberta, Canada; 3 Department of Medicine, Division of Geriatric Medicine, Dalhousie University, Halifax, Nova Scotia, Canada

**Keywords:** Alzheimer’s disease, amnestic mild cognitive impairment, data-driven analytics, deficit burden, frailty, health transitions, multimorbidity

## Abstract

**Background:**

Data-driven examination of multiple morbidities and deficits are informative for clinical and research applications in aging and dementia. Resulting profiles may change longitudinally according to dynamic alterations in extent, duration, and pattern of risk accumulation. Do such frailty-related changes include not only progression but also stability and reversion?

**Objective:**

With cognitively impaired and dementia cohorts, we employed data-driven analytics to (a) detect the extent of heterogeneity in frailty-related multimorbidity and deficit burden subgroups and (b) identify key person characteristics predicting differential transition patterns.

**Methods:**

We assembled baseline and 2-year follow-up data from the National Alzheimer's Coordinating Center for amnestic mild cognitive impairment (aMCI) and Alzheimer's disease (AD) cohorts. We applied factor analyses to 43 multimorbidity and deficit indicators. Latent Transition Analysis (LTA) was applied to the resulting domains in order to detect subgroups differing in transition patterns for multimorbidity and deficit burden. We characterized heterogeneity in change patterns by evaluating key person characteristics as differential predictors.

**Results:**

Factor analyses revealed five domains at two time points. LTA showed that two latent burden subgroups at Time 1 (*Low*, *Moderate*) differentiated into an additional two subgroups at Time 2 (adding *Mild, Severe*). Transition analyses detected heterogeneous changes, including progression, stability, and reversion. Baseline classifications and transitions varied according to clinical cohort, global cognition, sex, age, and education.

**Conclusions:**

Heterogeneous frailty-related subgroup transitions can be (a) detected in aging adults living with aMCI and AD, (b) characterized as not only progression but also stability and reversion, and (c) predicted by precision characteristics.

## Introduction

Frailty is a broad and multidimensional construct representing a dynamic accumulation and changing expression of multiple morbidities and deficits, including clinical conditions, symptoms, medical signals, physiological functioning, health behaviors and beliefs. Within community-dwelling aging populations, the exact profiles of morbidities and deficits contributing to frailty burden may vary across persons at any point in time and may change within-persons over time.^1^ Accordingly, when constituent components of frailty are assessed repeatedly over time in groups of cognitively unimpaired older adults, the observed changes may be variable and characterized by multidirectional patterns of deficit accumulation or dispensation.^2–5^ When examined longitudinally, such transitions prominently include progression of burden, but also, to a lesser-known degree, improvement or reversion^6^ and possibly longer-term recurrent fluctuations.^7–9^ Frailty is a well-known risk factor for such later adversities as cognitive impairment^10^ or dementia.^11^ However, it is unknown whether the cumulative burden of multiple morbidities and deficits is so consolidated in persons living with these conditions that differential transitions are minimized or clinically inconsequential.^12^ We examine whether such differential transitions continue to occur in a clinically impaired cohort and the extent to which the fluctuations assume multiple patterns of change.

A promising set of longitudinal studies have successfully modeled unidimensional frailty trajectories and transitions in cognitively impaired or dementia cohorts using an overall summary score representing multisystem deficit accumulation^13^ or physical phenotypes.^14,15^ For example, Yuan and colleagues assembled three waves of data (spanning a 6-month period) for older nursing home residents, a subset of whom had a baseline diagnosis of moderate or severe cognitive impairment.^13^ Participants completed the 7-item FRAIL-NH scale^16^ which includes markers of fatigue, resistance, ambulation, incontinence, weight loss, nutritional status, and help with getting dressed. Five distinct change trajectories were detected: *Consistently Robust* (prevalence: 4.8%)*, Improving Frailty* (5.5%), *Consistently Pre-Frail* (29%), *Consistently Frail* (53%), and *Worsening Frailty* (7.6%). The following characteristics were associated with an increased risk for the latter three trajectories: older age, female sex, minority racial/ethnic backgrounds, higher levels of cognitive impairment, and neurodegenerative disease (e.g., Parkinson's). Complementary research evaluated transitions across physical frailty phenotypes in 122 older adults with mild cognitive impairment (MCI) or mild-to-moderate Alzheimer's disease (AD).^14^ Participants contributed baseline and 1-year follow-up data for self-reported exhaustion, low energy expenditure, slow gait speed, and weak grip strength. Findings showed that 36% of the sample evinced stability (no additional deficits accumulated), 32% progressed (≥ 1 deficit accumulated), and 32% improved (≥ 1 deficit remediated). Age, sex, racial/ethnic background, global cognitive function, education, and Apolipoprotein E (*APOE*) ɛ4 + allele carrier status did not vary across transition patterns. These studies suggest that frailty can change in multiple directions in persons living with MCI or AD, including the possibility of reversion from more to less frailty.

Several reviews have expressed that an important direction of research is to evaluate whether there are identifiable frailty-related components (or domains) that are associated with multidirectional transition patterns.^17–19^ Some prior work has modeled dynamic alterations in selected domains (e.g., pain,^20^ depressive symptoms)^21^ using cognitively impaired^20–23^ or dementia^24^ cohorts. The current study is the first to our awareness to use Latent Transition Analysis (LTA) to distinguish and track latent subgroups representing multiple domains of morbidity and deficit burden in persons living with amnestic MCI (aMCI) or AD.

A strength of the LTA approach is that it can track and model heterogeneous and discontinuous changes^25^ in salient domains of multidimensional constructs (such as frailty)^18^ which may unfold differentially over time. Accordingly, LTA represents an important complement to alternative approaches (e.g., latent growth curve analysis) that are designed to model average linear trajectories of unidimensional developmental phenomena. Specifically, LTA is a data-driven mixture-modeling approach that can (a) identify unobserved subgroups across multiple time points (i.e., homogenous clusters of persons characterized by a similar pattern of multimorbidity and deficit burden), (b) model multidirectional patterns of latent subgroup transitions (stability, progression, reversion), and (c) elucidate precision predictors of baseline subgroup classifications and transitions.^26^ Regarding the latter consideration, the present study examines the following clinically relevant precision predictors:^6,12,27^ clinical cohort, global cognition, *APOE* ɛ4 + allele carrier status, race/ethnicity, chronological age, sex, and education. Findings from this study may reveal the extent of variability in pathways of multimorbidity and deficit burden in an impaired population, as well as key person characteristics that could be harnessed for promotion of healthier change patterns or prevention of related adverse outcomes (e.g., hospitalization).

### Research goals

Two research goals (RG) are specified. The first RG is comprised of two sequential (but related) analytic subgoals. For RG1a, we apply exploratory and confirmatory factor analyses to a longitudinal battery of multimorbidity and deficit items contributed by the combined clinical cohort (aMCI, AD). We expected these data-driven analyses to reduce the items into key observed domains of deficits for estimation feasibility in the subsequent LTA. For RG1b, we apply LTA to the resulting empirically-derived domains in order to (a) detect latent multimorbidity and deficit burden subgroups at baseline and 2-year follow-up and (b) characterize transition patterns. We expected that, within each of the two time points, this data-driven mixture-modeling approach would detect two or more subgroups differentiated by the pattern and severity of accumulated burden in the empirically-derived domains. In addition, we expected to observe multidirectional transitions across the latent subgroups identified within the two time points, such that detectable participant clusters would evince progression, stability, or reversion. For RG2, we test key person characteristics as predictors of baseline latent subgroup classifications and longitudinal transitions. We expected results would reveal significant effects for clinical cohort, global cognition, *APOE*, race/ethnicity, chronological age, sex, or education.

## Methods

### Participants

Data were drawn from the Uniform Data Set (UDS) of the National Alzheimer's Coordinating Center (NACC). To date, 39 Alzheimer Disease Centers (ADC) across the United States have contributed harmonized, large-scale, longitudinal data to the UDS. Participants are recruited (primarily through convenience sampling methods) to the UDS and followed approximately annually. Cognitive status is determined by ADC clinicians based on prevailing diagnostic research criteria.^28–30^ Informed consent is provided at the individual ADCs, as approved by individual Institutional Review Boards (IRB). The University of Washington's IRB approved the sharing of de-identified data from the UDS.^31^

The current study appeared in an earlier version as part of a doctoral dissertation.^32^ Specifically, we assembled data for all UDS visits conducted between January 2005 to March 2020. We developed firm rules of inclusion and exclusion and applied them to the full UDS (*n *= 42,661) in a series of data selection steps (see Supplemental Figure 1). We then stipulated five fundamental requirements for our research sample: (a) time points (restricted to participants with a minimum of three waves); (b) follow-up (restricted to in-person visits); (c) inter-wave intervals (restricted to intervals within +/- 1 SD from the overall interval mean); (d) clinical cohort (restricted to participants meeting the clinical criteria for MCI or dementia at baseline); and (e) age (restricted to participants aged ≥ 53 years at baseline). The latter criterion cut-off (a) captured 97% of the sample and (b) converges with previous research examining concordant research questions,^14^ including recent studies conducted using the UDS^33,34^ and related mixture-modeling analytics.^35^

We then disaggregated the sample by baseline clinical cohort and applied the following exclusionary criteria across the first three measurement occasions. Participants were excluded if they had: (a) diagnoses of cognitive impairment other than single- or multi-domain aMCI and (b) primary etiologic diagnoses of dementia due to a source other than AD. We then applied the following exclusionary criteria at baseline: (a) dementia severity other than mild or moderate (based on the Clinical Dementia Rating Scale^®^); (b) living situation other than private residence or retirement community; (c) psychiatric disorders (obsessive compulsive disorder, bipolar disorder, schizophrenia); (d) self-reported history of traumatic brain injury; and (e) race/ethnicity other than non-Hispanic White or Black/African American (due to the limited number of participants who self-identified as belonging to other racial/ethnic subgroups). We present baseline descriptive and clinical characteristics for the final study sample (*N *= 3074; *M*age = 74.70, range = 53–100 years; 52% female; 91% non-Hispanic White) in Table 1.

**Table 1.** Time 1 participant demographic and clinical characteristics.

Characteristic	Total sample	aMCI	AD	Sig.
*n*(%)	3074	878 (29%)	2196 (71%)	
*n*(%) self-reported female	1603 (52%)	411 (47%)	1192 (54%)	***
Age (y)	74.70 (8.69)	74.53 (7.52)	74.76 (9.12)	*^ns^*
Education (y)	15.08 (3.37)	16.08 (3.18)	14.68 (3.36)	***
*n*(%) non-Hispanic White	2782 (91%)	802 (91%)	1980 (90%)	*^ns^*
MMSE ^a^	23.28 (4.75)	26.96 (2.47)	21.89 (24.64)	***
CDR^®^	0.79 (0.42)	0.50 (0.10)	0.91 (0.44)	***
*n*(%) *APOE* ε4 carriers ^b^	1536 (58%)	395 (53%)	1141 (60%)	**
*n*(%) married	2215 (72%)	639 (73%)	1576 (72%)	*^ns^*
*n*(%) in private residence	2795 (91%)	806 (92%)	1989 (91%)	*^ns^*
Inter-wave interval (in days)	780.01 (78.88)	788.48 (79.69)	776.62 (78.32)	***
43-item frailty index (Time 1) ^c^	0.23 (0.12)	0.15 (0.09)	0.27 (0.12)	***
Range:	0–0.74	0–0.46	0–0.74	
43-item frailty index (Time 2) ^c^	0.32 (0.15)	0.20 (0.12)	0.36 (0.14)	***
Range:	0–0.86	0–0.65	0.03–0.86	

Results are presented as mean (standard deviation) or *n*(%) of the sample with the associated characteristic. *p-*values are based on independent sample *t*-tests or chi-square tests, as appropriate. aMCI: amnestic mild cognitive impairment; AD: Alzheimer's disease; Sig: significance; MMSE: Mini-Mental State Exam; CDR^®^: Clinical Dementia Rating Scale; *APOE*: Apolipoprotein E; *ns*: not significant. ^a^ Select participants were administered the Montreal Cognitive Assessment (MoCA) in lieu of the MMSE; education-adjusted scores on the MoCA were converted to an equivalent MMSE score using published conversion tables derived from the National Alzheimer's Coordinating Center Uniform Data Set.^40^ ^b^ Results are based on 746 participants with aMCI and 1903 participants with AD who were genotyped. ^c^ Higher values represent increasing levels of global frailty; values ≥ 0.21 can be used to assign frailty status.^63^

*** *p *< 0.001 ** *p *< 0.01.

### Measures

*Multimorbidity and deficit items.* We compiled three waves of data for a battery of 43 multimorbidity and deficit items. These items were chosen from the UDS based on validated procedures for assembling frailty-related indicators from an existing epidemiological dataset.^36^ Accordingly, each of the 43 items has been employed in large-scale longitudinal frailty research,^11,23,35,37,38^ including recent studies conducted using the UDS.^10,33,34^ Procedures for collecting these data included self-report, clinician examinations, and formal tests with standardized scales. Each item was coded for values ranged between 0 (no deficit) and 1 (deficit maximally expressed; details in Supplemental Table 1).^36^ Data-checking analyses indicated that there was limited change across many of these items from the first to second measurement occasion. We reasoned that this may be due in part to the relatively close spacing of the study intervals (*M* interval = 394.44 days; *SD *= 58.40). We opted to apply all subsequent analyses to data from the first and third measurement occasion (hereafter referred to as Time 1 and 2, respectively). The mean interval separating the first and second time point is 780.01 days (range = 575–1156 days; *SD *= 78.88 days). This duration of follow-up aligns with previous frailty-related mixture-modeling research using cognitively impaired cohorts^23^ and is ideal for detecting multidirectional transition patterns.^7^

*Precision predictor variables.* We assembled Time 1 data for the following key person characteristics: clinical cohort (0 = aMCI, 1 = AD; based on the prevailing clinical diagnostic criteria),^28–30^ global cognitive function (continuous performance on the Mini-Mental Status Exam (MMSE)^39^ or an equivalent measure),^40^ self-reported race/ethnicity (0 = non-Hispanic White, 1 = Black/African American), chronological age (in years), self-reported sex (0 = male, 1 = female), and educational background (years of formal schooling). A subset of participants (*n *= 2719) submitted blood samples to the NACC for genotyping. For these individuals, we assembled data pertaining to *APOE* ε4 allelic status (non-carrier = 0, carrier = 1). Because previous research indicates that the ε2 allele represents a protection factor and the ε4 allele represents a risk factor,^41,42^ participants with the ε2/ε4 genotype (*n *= 70) were excluded from the corresponding analyses. Descriptive statistics are presented in Table 1.

*43-item frailty index.* We operationalized an independent 43-item frailty index (FI) using the initial pool of multimorbidity and deficit items (Supplemental Table 1). Each constituent item adheres to standard construction guidelines for creating a valid and reliable FI.^36^ Accordingly, previous large-scale epidemiological research has operationalized an FI using concordant items contributed by cognitively unimpaired, impaired, and dementia cohorts.^11,36,43,44^ Continuous values on the 43-item FI were calculated for each participant by summing the values of all constituent items and then dividing by the total number of deficits (*N = *43).^36^ Values on the 43-item FI could thus range between 0 (no deficits endorsed) and 1 (all 43 deficits endorsed). Importantly, these values were calculated solely for two characterization purposes: (a) to provide background information about the participants and (b) to supplement (in planned post-hoc analyses) the interpretive labels assigned to the subgroups detected in the LTA. Descriptive statistics for the 43-item FI disaggregated by baseline clinical cohort and time point are presented in Table 1.^36^

### Analytical approach

Analyses were conducted using Mplus 8.0.^45^ A small proportion of participations were missing data for global cognitive function (*n *= 75; 2.4%) or educational background (*n *= 7; 0.2%). We estimated these missing data using multiple imputations. Specifically, we generated 20 imputations of the dataset and pooled these for all prediction analyses.^46–48^ Missing data for multimorbidity and deficit indicators were assumed to be completely at random (i.e., item nonresponse)^48^ and were handled using full-information maximum likelihood.

*RG1a: Detecting salient domains of deficit using exploratory and confirmatory factor analyses.* We separately applied (a) exploratory factor analysis (EFA) to the initial battery of 43 multimorbidity and deficit items at Time 1 and (b) confirmatory factor analysis (CFA) to the resulting latent structure at each time point. This data-driven approach reduced the number of indicators for estimation feasibility in the subsequent LTA (RG1b) by identifying empirically salient domains of deficits.^35^ We first applied EFA to Time 1 data contributed by a random subset (i.e., 50%) of the overall study sample. Decisions regarding the number of domains and indicators to retain were made in accordance with best-practices literature.^49,50^ We then verified that this latent structure fit the data at each time point (separately) by applying CFA to data contributed by (a) the remaining subset of participants (50%) and (b) the overall study sample. Model fit was determined using standard indices (see Supplemental Methods). Once the best-fitting model was established, we calculated the proportion (or relative number) of deficits participants had accumulated in each of the EFA-derived domains, at both time points. Values for each domain could range between 0 (none of the constituent deficits endorsed) and 1 (all of the constituent deficits endorsed). The proportion of deficits accumulated in the EFA-derived domains (at both time points) served as continuous observed indicators in the subsequent LTA (RG1b).

*RG1b: Applying LTA to the EFA-derived domains*. Two options for the LTA were considered (i.e., full clinical cohort approach or a multiple-group approach). We selected the former for two reasons. First, we checked the clinical status reversion rate for aMCI over the 2-year interval. We observed that, of the 878 participants with an initial diagnosis of aMCI, 366 (42%) were clinically classified at follow-up as having progressed to AD and 64 (7%) were clinically classified as having reverted to cognitively unimpaired status. This pattern is to be expected given the transitional (and potentially reversible) nature of aMCI. Accordingly, we elected to include these participants in the LTA. However, in a typical multiple-group LTA, the grouping variable does not vary across the study duration.^51^ Second, we conducted a series of stratified LTA in which we systematically disaggregated the data by the following Time 1 characteristics: clinical cohort (aMCI, AD), race/ethnicity (non-Hispanic White, Black/African American), sex (female, male), and age (young-old (< 75 years), old-old (≥ 75 years)). The results of all tested stratification models produced evidence of non-convergence or non-invariance of the longitudinal indictors. Therefore, consistent with prevailing conventions,^52^ we applied LTA to the EFA-derived domains in four sequential phases.

First, we applied a separate latent profile analysis to the deficit domains at each time point.^53^ This entailed fitting a sequence of models with varying numbers of subgroups (e.g., 1, 2, 3). We selected the best-fitting model by considering interpretability of the results, together with standard model parameters, tests, and fit indices (as described in Supplemental Methods).^54^ Entropy was not used for model selection, but rather to infer the accuracy with which participants were classified into the latent subgroups (ranges between 0–1; the higher the better). To avoid local maxima, we used 5000 multiple starting values at each time point. Indicators were allowed to covary within subgroups while the variance-covariance structures were constrained to equality across subgroups (i.e., class invariant-unrestricted structure).^54^ This structure provided a better fit to the data as compared to alternative models that allowed free estimation of variance-covariance structures across subgroups (i.e., class-varying unrestricted structure; results not reported).

Second, we examined longitudinal measurement invariance of the subgroups by evaluating the following sequence of similarity tests:^55,56^ (a) configural similarity, which tests whether the same number of latent subgroups based on the same indicators can be identified over time; (b) structural similarity, which tests whether within-subgroup indicator means are the same over time; (c) dispersion similarity, which tests whether within-subgroup variances (i.e., heterogeneity of the latent subgroups) are the same over time; and (d) distributional similarity, which tests whether the relative size of the subgroups are the same over time. We tested the tenability of invariance assumptions by comparing models with unconstrained and constrained parameters using -2 log-likelihood difference statistic.^51^ Empirical support for structural invariance permits two important observations regarding the subgroups observed at both time points.^26^ First, constraining the within-subgroup indicator means to equality over time permits the substantive interpretation (i.e., statistical characteristics and thus qualitative interpretation) of the Time 1 subgroups to generalize to their corresponding subgroup at Time 2. Second, participants who demonstrate statistically significant deficit accumulation or reduction in the EFA-derived domains are classified into a different subgroup at Time 2. Similarly, participants who show no significant changes in the EFA-derived domains are classified into the corresponding subgroup at follow-up. Accordingly, structural invariance permits researchers to explicitly evaluate the respective change patterns and reliably interpret them as representing progression, reversion, and stability.^26^

Third, we interpreted the best-fitting constrained (or invariant) model using an adapted formula for Cohen's *d*. Specifically, this formula was used to (a) calculate standardized mean differences across subgroups in each of the EFA-derived domains and (b) facilitate qualitative interpretation of the detected multimorbidity and deficit burden subgroups.^35,54^ Standardized mean differences > 2.0 indicate a less than 20% overlap in subgroup-specific distributions and a high degree of separation in the corresponding frailty-related domain, whereas values < 0.85 indicate more than 50% overlap and a low degree of separation.

Fourth, we examined multidirectional patterns of latent subgroup transitions (progression, stability, reversion) using the probabilities estimated in the final LTA solution. These values reflect the probability of being in a given subgroup at Time 2, conditional on being in a given subgroup at Time 1.

*RG2: Precision prediction analyses.* Participants were classified into their best-fitting latent subgroup at each time point^57–59^ using estimated posterior probabilities. We applied precision prediction analyses to these data in two phases. In phase one, we used logistic regression to evaluate the key person characteristics as differential predictors of Time 1 subgroup classifications. Because only a subset of participants had data available for *APOE* ε4 allelic status and published attempts to recover missing genes across an entire dataset remain limited,^60^ we performed a separate logistic regression in which we tested *APOE* together with the remaining predictors. For the latter analysis, we report only the results for *APOE.* In phase two, we stratified the sample by Time 1 subgroup classifications and performed separate logistic regressions in which we examined whether transition patterns varied according to the key person characteristics.

## Results

The analytic workflow of RG1a, RG1b, RG2, and the corresponding results are summarized in Supplemental Figure 2.

### RG1a: Detecting salient domains of deficit using exploratory and confirmatory factor analyses

Results from the application of EFA to the initial battery of 43 multimorbidity and deficit items revealed that a solution comprised of five salient domains (or factors) best fit the data. In this solution, 10 of the initial 43 items were eliminated due to either low factor loadings or high cross-loadings (final *n* of constituent indicators = 33; see Table 2).^49,50^ The subsequent CFA verified that (a) this latent structure provided adequate-to-good fit to the data at both time points (Supplemental Table 2) and (b) the constituent indicators for each domain were characterized by strong factor loadings (Supplemental Figures 3 and 4). The following interpretive labels were assigned to the five EFA-derived domains based on common characteristics of the constituent indicators and previous naming conventions:^23,35,61,62^ *physical function* (*n* constituent indicators = 5), emotional well-being (*n* constituent indicators = 5), *behavioral disturbances* (*n* constituent indicators = 9), *instrumental health* (*n* constituent indicators = 10), and cardiovascular symptoms (*n* constituent indicators = 4). For each participant, the proportion (or relative number) of deficits accumulated in each domain (at both time points) was calculated by summing the values across the constituent indicators and dividing this by the total number of constituent deficits. Accordingly, values for each domain (at both time points) could range between 0 (none of the constituent deficits endorsed) and 1 (all of the constituent deficits endorsed). These data were treated as continuous observed indicators in the subsequent LTA (RG1b). We report longitudinal sample descriptives disaggregated by the five EFA-derived domains in Table 2.

**Table 2.** Multimorbidity and deficit items disaggregated by the five exploratory factor analysis derived domains (total n of constituent indicators = 33).

EFA-Derived Domain	Constituent Indictor^+^
Physical Function	Bowel incontinence
	Urinary incontinence
	Slowing of motor movements
	Tremor
	Walking changed not due to injury or arthritis
Time 1 *M* (*SD*)	0.08 (0.16)
Time 2 *M* (*SD*)	0.17 (0.23)
Emotional Wellbeing	Satisfied with life
	Often feel helpless
	Prefer to stay home
	Often get bored
	Dropped many activities and interests
Time 1 *M* (*SD*)	0.16 (0.21)
Time 2 *M* (*SD*)	0.16 (0.22)
Behavioral Disturbances	Disinhibition
	Irritability and/or lability
	Apathy and/or indifference
	Agitation and/or aggression
	Anxiety
	Depression and/or dysphoria
	Appetite: changes in weight or food preferences
	Engages in repetitive activities
	Nighttime behaviors
Time 1 *M* (*SD*)	0.26 (0.23)
Time 2 *M* (*SD*)	0.31 (0.25)
Instrumental Health	Difficulty traveling out of the neighborhood
	Difficulty remembering appointments
	Difficulty paying attention to TV programs
	Difficulty keeping track of current events
	Difficulty preparing a balanced meal
	Difficulty turning off the stove
	Difficulty playing a game of skill
	Difficulty shopping alone
	Difficulty assembling tax records
	Difficulty paying bills
Time 1 *M* (*SD*)	0.36 (0.28)
Time 2 *M* (*SD*)	0.56 (0.32)
Cardiovascular Symptoms	Total number of medications
	Hypercholesterolemia
	Hypertension
	Diabetes
Time 1 *M* (*SD*)	0.42 (0.28)
Time 2 *M* (*SD*)	0.48 (0.28)

At each time point, we calculated the proportion of deficits participants had accumulated in each of the EFA-derived domains. Values for each domain could range between 0 (none of the constituent deficits endorsed) and 1 (all of the constituent deficits endorsed). These data were treated as continuous observed indicators in the Latent Transition Analysis (LTA). *M*: mean; *SD*: standard deviation. ^+ ^For indicator coding see Supplemental Table 1.

### RG1b: Applying LTA to the EFA-derived domains

As depicted in Supplemental Figure 2, the four sequential phases of the LTA included (a) conducting a separate latent profile analysis at each time point, (b) evaluating longitudinal measurement invariance of the detected subgroups, (c) interpreting the latent subgroups in the best-fitting constrained model, and (d) examining multidirectional patterns of latent subgroup transitions (progression, stability, reversion). Results for each phase are discussed in turn below.

*Phase 1: Separate latent profile analysis at each time point.* A complete reporting of the results is provided in Supplemental Table 3. At Time 1, results indicated that a solution comprised of two latent subgroups provided the best fit to the data based on the following considerations. First, Akaike Information Criterion (AIC), Bayesian Information Criterion (BIC), and sample-size adjusted BIC (SABIC) decreased with the addition of the second latent subgroup, indicating significant improvement in model fit. Second, the adjusted Lo-Mendell-Rubin likelihood ratio test (LMR-LRT; *p *= 0.11) and adjusted Vuong-Lo-Mendell-Rubin likelihood ratio test (VLMR-LRT; *p *= 0.11) values indicated that this solution was not significantly improved upon by the addition of a third subgroup. Third, participants were classified into the two subgroups with a high degree of precision (entropy = 0.99). At Time 2, we determined that a solution comprised of four latent subgroups provided the best fit to the data based on the following considerations. First, AIC, BIC, and SABIC all steadily decreased as the number of subgroups increased between one and four, indicating successive improvements in model fit. Second, the adjusted LMR-LRT (*p *= 1.0) and VLMR-LRT (*p *= 1.0) indicated that this solution was not significantly improved upon by the addition of a fifth subgroup. Third, participants were classified into the four subgroups with a high degree of precision (entropy = 0.91). This value is similar to the corresponding value observed at Time 1 (for a two-subgroup solution), collectively indicating that (a) the subgroups are well distinguished and (b) there is a high degree of certainty in participants’ best-fitting subgroup classification.

*Phase 2: Longitudinal measurement invariance tests.* Full results from the longitudinal measurement invariance tests are available in Supplemental Table 4. Regarding configural and distributional similarity, results from the latent profile analysis indicated that a different number of subgroups provided the best fit to the data at each time point. Accordingly, we did not find evidence in support of configural or distributional invariance (no formal tests were required). This pattern of results (a) is often observed in developmental and aging research^51^ and (b) reflects increased heterogeneity in the pattern of multimorbidity and deficit burden from the first to second time point. We subsequently evaluated structural invariance of the two subgroups observed at both Time 1 and Time 2 by estimating two nested models. In the first model, the within-subgroup indicator means for the five EFA-derived domains were free to vary at Time 2. In the second (structurally invariant) model, we constrained the within-subgroup indicator means for the five EFA-derived domains to be equal to the corresponding deficit domain at Time 2. These constraints significantly improved model fit. Next, we evaluated dispersion invariance by estimating a third model. In this model, we constrained the five within-subgroup indicator variances to equality at Time 2. Model fit significantly worsened with the addition of these constraints. Accordingly, we elected to retain and interpret the second model, which is comprised of the two structurally invariant subgroups, as well as the two emerging subgroups (i.e., those that were statistically evident or detectable at Time 2).

**Figure 1.**
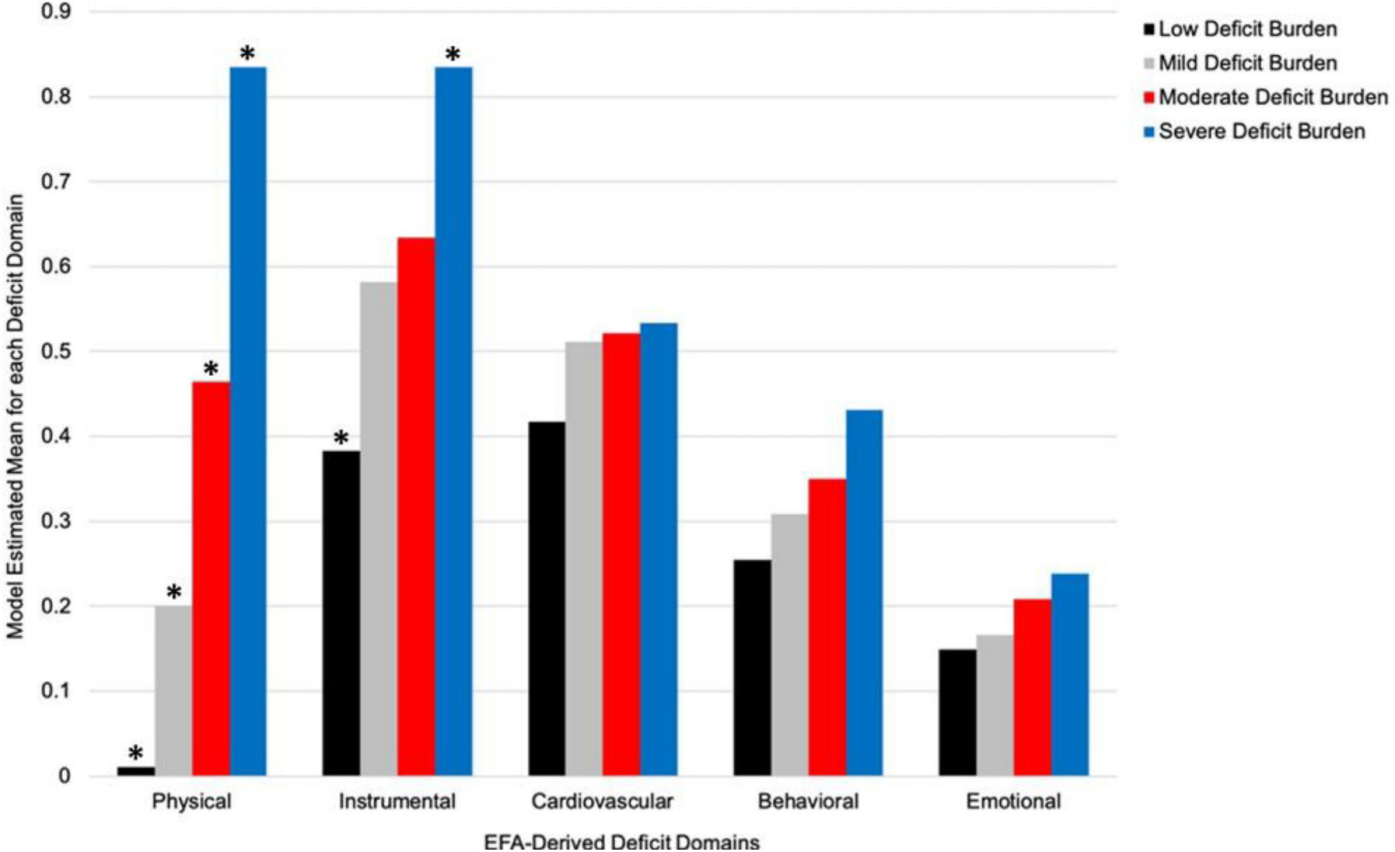
Model estimated means for the five EFA-derived domains disaggregated by the Time 2 latent subgroups. The latent subgroups were ordered along a continuum of increasing deficit burden across domains. The subgroup differences are most marked for physical function and instrumental health, which appear to be the principal drivers of the Severe Deficit Burden subgroup. The cardiovascular symptoms domain is notable for the tight cluster of intermediate values evident in all four subgroups. *Denotes model estimated means characterized by a moderate-to-high degree of separation between the corresponding subgroups (See Supplemental Table 5 for standardized mean differences). Physical: physical function; Instrumental: instrumental health; Cardiovascular: cardiovascular symptoms; Behavioral: behavioral disturbances; Emotional: emotional well-being.

*Phase 3: Labeling the latent subgroups.* We next assigned interpretive labels to the latent subgroups using the observed pattern of standardized mean differences across the five EFA-derived domains (Supplemental Table 5). We characterized the two subgroups observed at both time points as *Low Deficit Burden* and *Moderate Deficit Burden*. At Time 1, these subgroups were distinguished by the level of multi-domain deficit accumulation (particularly physical function) such that the *Low Deficit Burden* subgroup (*n *= 2790; 91%) was characterized by a lower burden relative to the *Moderate Deficit Burden* subgroup (*n *= 284; 9%). At Time 2, our results showed that (a) the *Low Deficit Burden* (*n *= 1714; 56%) and *Moderate Deficit Burden* (*n *= 571; 19%) subgroups not only had distribution-related changed prevalence, but (b) had differentiated into two emerging subgroups that we characterized as *Mild Deficit Burden* (*n *= 654; 21%), a new intermediate level of multi-domain deficit accumulation, and *Severe Deficit Burden* (*n *= 153; 4%), representing substantial progression in adversity. As can be seen in Figure 1, the four latent subgroups are ordinally distributed according to severity of accumulated burden within each of the five EFA-derived domains. This is especially evident for the domains representing physical function and instrumental health.

*Supplementing the latent subgroup interpretations: Post-hoc analysis of the 43-item FI.* The observed pattern of subgroup differences on the independently calculated 43-item FI buttressed the interpretive labels assigned to the latent subgroups. Specifically, at Time 1, the Low Deficit Burden subgroup was characterized by lower FI values (*M *= 0.22; *SD *= 0.12) relative to the Moderate Deficit Burden subgroup (*M *= 0.37; *SD *= 0.13). Similarly, at Time 2, we found that values on the 43-item FI increased successively across the latent subgroups (*F*(3, 3051) = 377.31, *p *< .001): Low Deficit Burden (*M *= 0.26; *SD *= 0.13), Mild Deficit Burden (*M *= 0.33; *SD *= 0.13), Moderate Deficit Burden (*M *= 0.43; *SD *= 0.13), and Severe Deficit Burden (*M *= 0.55; *SD *= 0.14). We emphasize that the 43-item FI was not part of the LTA but contributed valuable post-hoc information about the latent subgroups, including distributional variations in FI values (Supplemental Table 6). Accordingly, a subset of participants classified into the Low Deficit Burden subgroup at either time point were characterized by 43-item FI values that fell near or above the clinical threshold typically used to assign frailty status.^63^

*Are the baseline aMCI and AD cohorts differentially represented in the latent subgroups?* We evaluated whether participants with an initial diagnosis of aMCI or AD were differentially represented in the latent subgroups using follow-up χ^2^ tests and logistic regression. The former analyses indicated that at Time 1 (χ^2^_(1, 3074) _= 23.45, *p *< 0.001): (a) a larger proportion of the aMCI cohort was classified into the Low Deficit Burden subgroup (aMCI cohort proportion = 95%; AD cohort proportion = 89%) and (b) a larger proportion of the AD cohort was classified into the Moderate Deficit Burden subgroup (aMCI = 5%; AD = 11%). Similarly, at Time 2 (χ^2^_(3, 3074) _= 104.98, *p *< 0.001): (a) a larger proportion of participants with an initial diagnosis of aMCI were classified into Low Deficit Burden (aMCI = 69%; AD = 50%) and (b) a larger proportion of the initial AD cohort was represented in the remaining three subgroups, Mild (aMCI = 18%; AD = 22%), Moderate (aMCI = 11%; AD = 22%), and Severe (aMCI = 2%; AD = 6%). Logistic regression analyses showed that clinical cohort (i.e., an initial diagnosis of aMCI or AD) independently predicted latent subgroup classifications at each time point. Specifically, at Time 1 (χ^2^_(1) _= 25.95, *p *< 0.001), persons with a baseline diagnosis of AD were on average 2.20 (95% confidence interval [CI] = 1.59–3.05, *p* < .001) times more likely to be classified into Moderate Deficit Burden (relative to Low Deficit Burden) as compared to those with a baseline diagnosis of aMCI. At Time 2 (χ^2^(3) = 112.41, *p *< 0.001), persons with a baseline diagnosis of AD were more likely than those with aMCI to be classified into the Mild (odds ratio [OR] = 1.63, 95% CI = 1.33–2.00, *p *< 0.001), Moderate (OR = 2.81, 95% CI = 2.20–3.59, *p *< 0.001), or Severe subgroups (OR = 4.38, 95% CI = 2.54–7.55, *p *< 0.001) relative to Low Deficit Burden. We present additional comparisons in baseline descriptive and clinical characteristics (e.g., continuous MMSE values) across the Time 1 and Time 2 subgroups in Supplemental Table 6.

**Figure 2.**
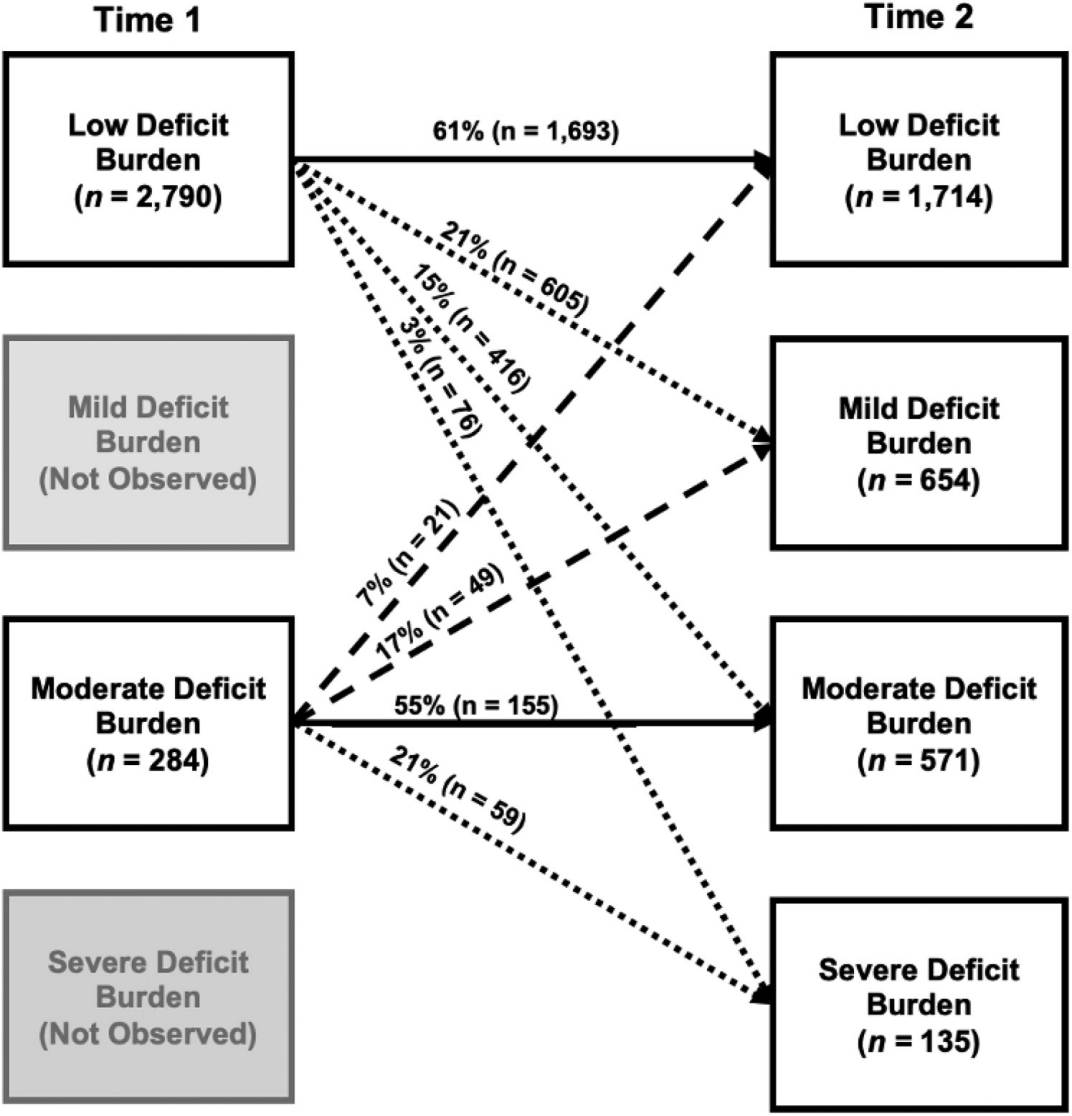
Unconditional latent transition probabilities disaggregated by the Time 1 subgroups. Two subgroups were observed at Time 1, Low Deficit Burden and Moderate Deficit Burden. These differentiated into an additional two observed subgroups at Time 2, Mild Deficit Burden and Severe Deficit Burden. Arrows between Time 1 and Time 2 represent all observed latent subgroup transitions. All arrows are labelled with the computed unconditional latent transition probability and number of participants transitioning in this direction. Solid lines represent observed stability (i.e., classification into the same subgroup at follow-up). Dotted lines represent observed progression (i.e., classification into a later subgroup characterized by higher multimorbidity and deficit burden). Broken lines represent observed reversion (i.e., classification into a later subgroup characterized by lower multimorbidity and deficit burden).

*Phase 4: Examining multidirectional patterns of latent subgroup transitions.* In Figure 2, we display unconditional latent transition probabilities separately for the two Time 1 subgroups, Low Deficit Burden and Moderate Deficit Burden. Our examination of these results is informed by the expectation of observing the following transition patterns across the two time points: stability (i.e., classification into the same subgroup at both time points), progression (i.e., classification into a later subgroup that is characterized by higher accumulated burden in the EFA-derived domains), and reversion (i.e., classification into a later subgroup that is characterized by lower burden in the EFA-derived domains). The latter two patterns (progression, reversion) can be used to characterize persons who transition to (a) a neighbouring subgroup or (b) a more distal (i.e., not neighbouring) subgroup.

For the Time 1 Low Deficit Burden subgroup (*n *= 2790; 91%), results indicated that most (*n *= 1693; 61%) remained classified as such at Time 2 (i.e., probability of stability = 0.61). Correspondingly, of the 39% (*n *= 1097) who appeared in a different subgroup at Time 2, most constituted the adjacent subgroup, Mild Deficit Burden (*n *= 605; 21%). However, 15% (*n *= 416) of this initial subgroup populated Moderate Deficit Burden at follow-up, with a smaller representation in the Severe subgroup (*n *= 76; 3%). Findings for the Time 1 Moderate Deficit Burden subgroup (*n *= 284; 9%) showed that approximately half (*n *= 155; 55%) remained classified as such at Time 2 (i.e., probability of stability = 0.55). Of the initial Moderate Deficit Burden subgroup who appeared in a different subgroup at Time 2 (*n *= 129; 45%), 21% (*n *= 59) populated the subgroup reflecting the most advanced level of deficit accumulation in the five EFA-derived domains, Severe Deficit Burden. However, a substantial portion of this initial subgroup constituted the Mild (*n *= 49; 17%) or Low Deficit Burden (*n *= 21; 7%) subgroups at follow-up.

### RG2: Precision prediction analyses in two phases

As depicted in Supplemental Figure 2, the two sequential phases included evaluating the key baseline person characteristics as differential predictors of (a) Time 1 latent subgroup classifications and (b) subsequent patterns of latent subgroup transitions (progression, stability, reversion).

*Phase 1: Predicting Time 1 latent subgroup classifications.* We present ORs from the binomial logistic regression in Table 3. Results indicated that risk for Time 1 Moderate Deficit Burden subgroup classifications was increased by (a) an initial diagnosis of AD (as compared to aMCI; OR = 1.79) and (b) each additional year of age (OR = 1.08). In contrast, risk for Time 1 Moderate Deficit Burden classifications was decreased by: (a) female sex (as compared to male sex; OR = 0.75) and (b) each unit increase in initial MMSE performance (representing global cognition; OR = 0.97). Time 1 latent subgroup classifications did not vary by the following person characteristics: race/ethnicity, educational background, or *APOE*.

**Table 3.** Odds ratios for predictors of Time 1 latent subgroup classifications.

Predictor	Odds Ratio [95% confidence interval]
Clinical cohort	1.79 [1.24, 2.57] **
Global cognition	0.97 [0.94, 0.99] *
Race/ethnicity	1.17 [0.78, 1.75]
Chronological age	1.08 [1.06, 1.09] ***
Self-reported sex	0.75 [0.58, 0.98] *
Education	1.00 [0.96,1.04]
*APOE* ε4 allele status ^a^	0.80 [0.60, 1.05]

The Low Deficit Burden subgroup was specified as the reference category. Baseline data for all predictors were entered simultaneously as predictors of Time 1 latent subgroup classifications: clinical cohort (0 = initial diagnosis of aMCI, 1 = initial diagnosis of AD), global cognitive function (continuous performance on the Mini-Mental Status Exam^39^ or an equivalent measure),^40^ self-reported race/ethnicity (0 = non-Hispanic White, 1 = Black/African American), chronological age (in years), self-reported sex (0 = male, 1 = female), education (years of formal schooling), *APOE* ε4 allele status (0 = non-carrier, 1 = carrier). aMCI: amnestic mild cognitive impairment; AD: Alzheimer's disease; *APOE*: Apolipoprotein E. ^a^ Results are based on the subset of participants who were genotyped (*n *= 2649).

***p < 0.001; **p < 0.01; *p < 0.05.

*Phase 2: Predicting multidirectional transition patterns.* We performed two multinomial logistic regressions (i.e., one for each Time 1 latent subgroup) to determine the leading predictors of differential change. We evaluated baseline data for the following predictors: clinical cohort, continuous MMSE performance, age, sex, and education. Race/ethnicity and *APOE* were excluded due to model non-convergence. Full results are presented in Table 4. Among those initially classified into the Low Deficit Burden subgroup, an initial diagnosis of AD (as compared to aMCI), older age, female sex (as compared to male sex), and higher education were associated with an increased likelihood of latent subgroup progression, whereas better initial performance on the MMSE increased the likelihood of latent subgroup stability. Among those initially classified into the Moderate Deficit Burden subgroup, younger age and better initial performance on the MMSE was associated with an increased likelihood of latent subgroup reversion.

**Table 4.** Odds ratios for predictors of subgroup transitions disaggregated by Time 1 latent classifications.

		Time 2 subgroup	
	Mild Deficit Burden	Moderate Deficit Burden	Severe Deficit Burden
Time 1 subgroup: Low Deficit Burden^+^			
Clinical cohort	1.49 [1.17, 1.90] ***	2.19 [1.60, 2.99] ***	2.52 [1.09, 5.86] *
Global cognition	0.99 [0.96, 1.01]	0.93 [0.91, 0.96] ***	0.86 [0.82, 0.90] ***
Chronological age	1.05 [1.04, 1.07] ***	1.07 [1.06, 1.09] ***	1.07 [1.04, 1.10] ***
Self-reported sex	1.22 [1.02, 1.51] *	0.91 [0.72, 1.14]	0.87 [0.54, 1.42]
Education	1.0 [0.97, 1.03]	1.05 [1.02, 1.09] **	1.01 [0.95, 1.08]

	Low Deficit Burden	Mild Deficit Burden	Severe Deficit Burden
Time 1 subgroup: Moderate Deficit Burden^+^			
Clinical cohort	0.68 [0.20, 2.34]	1.55 [0.57, 4.22]	0.72 [0.28, 1.94]
Global cognition	1.03 [0.93, 1.15]	1.08 [1.00, 1.17] *	0.96 [0.91, 1.02]
Chronological age	0.98 [0.92, 1.04]	0.95 [0.91, 0.99] *	0.99 [0.96, 1.04]
Self-reported sex	0.61 [0.22, 1.69]	1.05 [0.53, 2.07]	0.87 [0.46, 1.62]
Education	1.08 [0.91, 1.28]	0.96 [0.86, 1.06]	0.93 [0.85, 1.02]

^+^ For each model, the corresponding subgroup at Time 1 was specified as the reference category. All covariates were entered simultaneously as predictors of transition patterns: clinical cohort (0 = aMCI, 1 = AD), global cognitive function (continuous performance on the Mini-Mental Status Exam^39^ or an equivalent measure),^40^ chronological age (in years), self-reported sex (0 = male, 1 = female), and education (years of formal schooling). Odds ratios are presented together with the 95% confidence interval.

***p < 0.001; **p < 0.01; *p < 0.05.

*Can latent subgroup classifications and transitions be disaggregated from cognitive impairment (or severity of AD progression)?* Our analytical approach addressed a prominent challenge in the larger frailty-cognition literature; viz., disaggregating the reciprocal associations between multimorbidity (or deficit burden) and cognitive impairment (or severity of AD progression). Specifically, results from Phase 3 of the LTA demonstrated that all four latent subgroups were populated by both of the initial clinical cohorts (aMCI, AD). In Supplemental Table 7, we present results from a post-hoc analysis in which we compared the proportion of participants within each of the initial clinical cohorts that demonstrated progression, stability, or reversion of latent subgroup classifications (i.e., collapsing across the two Time 1 subgroups). The two cohorts differed significantly in terms of the proportion of participants who evinced stability (aMCI > AD) or progression (aMCI < AD), but did not differ in terms of the proportion of participants who demonstrated reversion. Although the latter result did not reach statistical significance, it is interesting to note that, of the 70 individuals who reverted latent subgroups, 56 (80%) had a baseline diagnosis of AD and 14 (20%) had a baseline diagnosis of aMCI. Taken together, these results indicate that a subset of the initial aMCI and AD cohorts were characterized by latent subgroup classifications and transitions patterns that differed from would be expected based on their cognitive status (or severity of cognitive impairment).

## Discussion

We assembled baseline and 2-year follow-up data for a diverse inventory of frailty-related morbidities and deficits in a large, well-characterized sample of persons with an initial diagnosis of aMCI or AD. For RG1a, we integrated a series of data-reduction techniques and data-driven analytics in order to identify salient domains within each time point. The first factor analyses revealed five multi-indicator domains of deficits: physical function, emotional well-being, behavioral disturbances, instrumental health, and cardiovascular symptoms. For RG1b, we submitted the five EFA-derived domains to LTA in order to detect (a) latent multimorbidity and deficit burden subgroups within each time point and (b) multidirectional patterns of latent subgroup transitions. For RG2, we identified precision predictors of (a) Time 1 subgroup classifications and (b) longitudinal transition patterns.

### RG1b: LTA in four phases

*Phases 1-3.* The data-driven latent profile analysis distinguished two subgroups at Time 1, including the predominant *Low Deficit Burden* (*n *= 2790; 91%) and a smaller *Moderate Deficit Burden* (*n *= 284; 9%). The latent profile analysis at Time 2 and subsequent longitudinal measurement invariance tests collectively indicated that (a) the statistical characteristics (and thus substantive interpretation) of the *Low Deficit Burden* (*n *= 1714; 56%) and *Moderate Deficit Burden* (*n *= 571; 19%) subgroups generalized across time points and (b) the two initial subgroups not only had distribution-related changed prevalence at follow-up, but (c) had differentiated into two emerging subgroups that were interpreted as *Mild Deficit Burden* (*n *= 654; 21%) and *Severe Deficit Burden* (*n *= 135; 4%).

Our qualitative interpretation of the detected subgroups was predicated on the pattern of standardized mean differences in the five EFA-derived domains. We supplemented these interpretations using post-hoc comparisons of the 43-item FI. Collectively, our findings showed that the Low, Mild, Moderate, and Severe Deficit Burden subgroups were ordered along a continuum of increasing multimorbidity and deficit burdens, as well as successively higher FI values. Interestingly, the domains most prominently driving the differentiation of latent subgroups were (a) physical function, which is characterized by indicators representing incontinence, tremor, and changes in motor movements or walking, and (b) to a slightly lesser extent, instrumental health, which is characterized by indicators representing dependence in instrumental activities of daily living (e.g., travelling out of the neighbourhood, shopping alone, paying bills). It is possible that the subgroups were primarily distinguished by these two domains (and not emotional well-being, behavioral disturbances, or cardiovascular symptoms) because the constituent indicators primarily reflect higher-order functions which rely on multiple systems working together in a coordinated and integrated fashion (e.g., musculoskeletal, nervous, cardiovascular, sensorial). As a result, deficits in physical function and instrumental health domains may account for variance (or integrate information) associated with numerous additional frailty-related domains.^35,61,64–68^

Previous data-driven research conducted with cognitively unimpaired cohorts has distinguished complementary subgroups.^35,69^ For example, Bohn and colleagues^35^ applied latent profile analysis to empirically-derived domains of deficits representing mobility, emotional well-being, instrumental health, cardiac symptoms, comorbidity, and respiratory symptoms. Three subgroups were detected— *not-clinically frail* (84%), *mobility-type frailty* (9%), and *respiratory-type frailty* (7%). The former subgroup was characterized by minimal multi-domain deficit burden, whereas the respective two subgroups were distinguished by marked burdens in mobility (and emerging impairment in instrumental health) and respiratory function. Other research assembled cross-sectional data for nursing home residents who ranged across multiple diagnostic categories of aging and neurodegeneration.^70^ Latent class analysis revealed *mild physical frailty*, *moderate physical frailty*, and *severe physical frailty*, each of which was distinguished by deficits in physical function (e.g., incontinence, inability to walk unassisted). These results, together with the present study findings, suggest that deficits in physical function and instrumental health are key defining characteristics of empirically-derived subgroups across the spectrum of cognitively unimpaired aging, including AD. Notably, mobility and related functional abilities (i.e., instrumental and basic activities of daily living) are also at the core of classifying individuals along fitness-frailty continua using the Clinical Frailty Scale.^68^ Overall, these results support the clinical relevance of the differentiating domains we observed and the subgroups we detected.

*Phase 4.* Notably, 61% of the combined clinical cohort who were initially classified into Low Deficit Burden populated this subgroup at the 2-year follow-up. Such stability in lower levels of deficit burden has been documented in cognitively unimpaired aging cohorts across longitudinal intervals ranging between 1 to 17 years.^3,4,71^ Our results show that this pattern may also characterize a substantial portion of cognitively impaired and dementia cohorts.^13–15,72^ This indicates that, although frailty and these clinical aging conditions frequently (and eventually) co-occur, persons living with diagnosed impairment or dementia may not inevitably present with (or quickly progress into) a phase of multi-domain deficit accumulation leading to classifiable and persistent global frailty. In line with this assertion, Burt and colleagues showed evidence of no frailty in 89% of a cross-sectional clinical cohort who spanned a range of neurodegenerative disorders, including MCI and AD.^37^ Similarly, a systematic review of frailty prevalence in mild-to-moderate AD reported that, on average, 68% of those living with this clinical condition were non-frail.^73^ Of the present Low Deficit Burden individuals who changed latent subgroups (39%), the most common transition was towards the neighbouring subgroup, Mild Deficit Burden (21%), indicating a relatively slow pattern of progression. A comparatively smaller proportion appeared in subgroups representing a marked increase in the severity of multi-domain deficit burden, including Moderate (15%) or Severe (3%).

Fifty-five percent of the initial Moderate Deficit Burden subgroup maintained 2-year stability. Among the 45% who appeared in a different latent subgroup at follow-up, 24% reverted to either Mild (17%) or Low Deficit Burden (7%), while the remaining 21% progressed towards the Severe subgroup. Several recent reviews have highlighted that spontaneous clinical remission of phenotypic or global frailty remains scarcely considered (in groups of cognitively unimpaired or impaired aging) and identified this as a priority area.^6,17^ Our results respond to this challenge by using longitudinal data for a well characterized clinical cohort to show that reversion (or reduction) of multimorbidity and deficit burden is possible among older adults living with aMCI or AD.^13–15^ Future large-scale epidemiological studies are encouraged to employ analytical techniques that elucidate multidirectional transition patterns, as well as associated mechanisms.

The pattern of progression from Moderate to Severe Deficit Burden suggests that deficits subsumed under the physical function domain may serve as a harbinger for adverse transition patterns for a subset of persons living with aMCI or AD. Complementary results have been documented with cognitively unimpaired older adults.^2,5,74–76^ For example, one study showed that performance on a rapid gait test predicted frailty phenotype transitions such that participants with poorer mobility were less likely to remain stable or improve.^2^ More recently, Bohn and colleagues^35^ showed that older adults with mobility-type frailty demonstrated the fastest rate of FI progression across a 40-year band of aging. The authors concluded that mobility-related deficits may function as portals to classifiable global frailty, which then cascades into more rapid and widespread deficit accumulation. We show that such deficits may also serve as morbidity-intensive portals to advancing multimorbidity and deficit burden in aMCI or AD.

### RG2: Precision prediction analyses in two phases

For RG2, we evaluated seven clinically relevant person characteristics as differential precision predictors of (a) Time 1 subgroup classifications (phase 1) and (b) patterns of 2-year subgroup transitions (phase 2). As discussed in turn below, significant effects were detected for the following baseline predictors: clinical cohort (i.e., initial diagnosis of aMCI as compared to AD), global cognition (i.e., continuous performance on the MMSE), sex (i.e., males as compared to females), chronological age (in years), and, to a lesser extent, education (i.e., years of formal schooling).

Regarding clinical cohort, we found that (a) persons with an initial diagnosis of aMCI were more likely to be classified into the Low Deficit Burden subgroup and (b) persons with an initial diagnosis of AD were more likely to be classified into the Moderate subgroup. Regarding longitudinal patterns of latent subgroup transitions, persons with a baseline diagnosis of AD were at an approximately two-fold increased risk for progressing from Low Deficit Burden to the Mild, Moderate, or Severe subgroups relative to those with aMCI, who were more comparatively more likely to remain stable. To our knowledge, this study is the first to show that these clinical cohorts are differentially associated with such latent subgroup classifications and transitions. Further research on multimorbidity and deficit burden transition characteristics is encouraged. For example, the present study focused on evaluating clinical cohort as a time-invariant predictor. About half of the initial aMCI cohort (*n *= 878) transitioned clinical status, with an expected small proportion reverting to cognitively unimpaired status (*n *= 64; 7%) and a larger proportion converting to an AD diagnosis (*n *= 366; 42%). Follow-up research could evaluate whether patterns of latent subgroup classifications and transitions vary as a function of clinical progression (or reversion) across gradations along the cognitively unimpaired to AD spectrum. Although we are not aware of previous work with such data-driven multimorbidity and deficit burden subgroups, we note that some longitudinal studies have addressed related issues regarding frailty and cognitive status changes or accumulating AD-related neuropathology.^11,14,77,78^ Similarly, growing literature has evaluated frailty as a predictor of incident MCI,^10,79^ dementia conversion,^34,80^ and AD.^38,81^ The reported associations harmonize with the current research in that higher deficit burdens exacerbated risk for these clinical conditions.

With respect to global cognition, we found that better baseline performance reduced risk for classification into the Moderate Deficit Burden subgroup at Time 1. In addition, better performance predicted an increased likelihood of (a) 2-year stability in the Low Deficit Burden subgroup and (b) reversion from the Moderate Deficit Burden subgroup, thus indicating a reduction in multimorbidity and deficit burden. More specifically, among those initially classified into the Low Deficit Burden subgroup, each unit increase on the MMSE (a) reduced the likelihood of progressing to the Moderate or Severe subgroups by 7–14% and (b) increased in the odds of reverting from Moderate Deficit Burden to Mild Deficit Burden by 8%. Other research has similarly reported that global cognition is associated with latent classifications in cognitively unimpaired aging cohorts. For example, older adults classified into a non-frail subgroup were characterized by better global cognition^69,76^ and/or an attenuated rate of cognitive decline^35,76^ relative to those classified as having mobility-type frailty. Yuan and colleagues showed that poorer performance on a brief cognitive screening measure predicted assignment into subgroups marked by poorer physical function^70^ or accelerated frailty progression.^13^ Our findings regarding the baseline clinical cohorts and global cognition collectively suggest that cognitive status and function mutually and variably interact with patterns of multi-domain deficit accumulation or dispensation.^82–84^

Regarding sex, we found females were more likely to be classified into the Low Deficit Burden subgroup at Time 1 and males were more likely to be classified into the Moderate subgroup. Patterns of latent subgroup transitions also varied by sex, whereby females were 22% more likely than males to progress from Low Deficit Burden to Mild Deficit Burden. Sex differences in data-driven frailty-related classifications and transitions are emerging areas of research interest.^13,69,76,85^ One study found that cognitively unimpaired males were overrepresented in a *fit* (or non-frail) latent subgroup, whereas females were overrepresented in subgroups characterized by mobility-related deficits.^86^ Other research reported that (a) membership in not-clinically frail and mobility-type frailty subgroups did not vary according to sex and (b) sex did not moderate the association between mobility-type frailty and FI progression.^35^ Lafortune and colleagues^23^ applied LTA to 17 multimorbidity and deficit items, the results of which revealed four subgroups that were interpreted as *cognitively and physically impaired*, *cognitively impaired*, *physically impaired*, and *relatively healthy*. In line with the present study, baseline prediction analyses indicated that males were more likely to be classified into the *physically impaired* subgroup, which was characterized by reduced mobility and functional limitations, whereas females were more likely to be classified as *relatively healthy*. Sex differences in transition patterns were also observed. *Relatively healthy* females were comparatively more likely than males to transition towards subgroups representing more compromised health states at a 2-year follow-up. We advance this line of investigation by demonstrating that males living with aMCI or AD initially presented with an increased risk for classification into an empirically-derived subgroup marked by greater multi-domain deficit burden. However, females were more likely than males to demonstrate progression in subgroup classifications.^13,14^ Future research could evaluate whether sex may confer differential risks for latent subgroup classification and transitions in cognitively unimpaired aging as compared to aging characterized by impairment or dementia. Increased understanding of sex differences in frailty-related transitions or trajectories may contribute to better identification of subgroups of older adults who would benefit from early and targeted interventions.

Regarding age, we found that each additional year was (a) associated with an 8% increased risk for classification into the Moderate Deficit Burden subgroup at Time 1, as well as (b) a 5–7% higher likelihood of progressing from Low Deficit Burden to the Mild, Moderate, or Severe subgroup. Interestingly, among those initially classified into the Moderate Deficit Burden subgroup, younger age predicted an increased likelihood of reverting to Mild Deficit Burden. Several studies have (separately) identified age as a risk or protection factor for adverse data-driven subgroup classifications, phenotype transitions, or FI trajectories in cognitively unimpaired, impaired, or dementia cohorts.^70,78,87,88^ However, this pattern has not been consistently reported.^27,89,90^ In fact, a recent systematic review concluded that the association between age and patterns of frailty progression or dispensation remains unclear and established this as a priority area of investigation.^12^ The present study addresses this research direction and identifies age as an important predictor of multidirectional patterns of multimorbidity and deficit burden in aMCI and AD.

Regarding education, we found that years of formal schooling was neither a risk nor protection factor for Time 1 subgroup classifications. Similarly, education was largely unassociated with transition patterns, with the exception that more years of formal schooling increased the likelihood of progressing from the Low to Moderate Deficit Burden subgroup. The reasons for the latter association are unclear, as related data-driven research has primarily controlled for education or not considered it as among one of the potential precision predictors.^4,23,35,69,70^ Education effects on frailty-related transitions or trajectories could be clarified in future research.

### Strengths and limitations

We note several methodological strengths and limitations. First, for strengths, we tracked the course of multimorbidity and deficit burden subgroups over 26 months using data-driven mixture-modeling techniques. The LTA approach includes (a) model-based participant classifications, (b) statistical diagnostic tools that elucidate the quality of participant classifications, (c) information-theoretic indices that promote selection of the most parsimonious model (thus discouraging overfitting),^91^ and (d) the capability to detect and model discontinuous (or non-linear) changes in multidimensional constructs.^25^ In these ways, LTA represents an important complement to alternative approaches to detecting homogenous subgroups (e.g., cluster analysis) and modeling change trajectories (e.g., latent growth curve analysis).

Second, we characterized and validated the detected latent subgroups by performing precision prediction analyses with features spanning clinical, cognitive, genetic, lifestyle, and demographic domains. In doing so, we addressed an important research direction highlighted in systematic reviews.^12,27^ Third, we advance understanding of key subgroup and person characteristics that may lead to differential patterns of multimorbidity and deficit burden, including progression, stability, and even reversion. We are unaware of any prior work that has evaluated these research directions in a combined clinical cohort of persons with living with aMCI or AD. Future replications and extensions are encouraged. For example, because the number of participants who self-identified into racial/ethnic subgroups other than non-Hispanic White or Black/African American was too small to permit statistically meaningful analysis, the present study focused on the two larger groups represented in our dataset: non-Hispanic White (91%) and Black/African American (9%). Follow-up work could evaluate whether the present findings generalize to older adults of diverse racial, ethnic, or Indigenous backgrounds. In addition, the present study focused on evaluating whether patterns of latent subgroup classifications and transitions varied according to continuous values on the MMSE. The latter practice (a) is consistent with previous multimorbidity^92–94^ and frailty research,^69,76^ including recent studies conducted using the UDS^10,33,34^ and (b) circumvents commonly encountered uncertainties related to optimal cut-off scores on the MMSE, thus increasing potential generalizability of the study findings.^95,96^ Follow-up studies could explore whether prediction patterns may vary across discrete levels of global cognitive function.

Regarding limitations, we first note that prediction analyses were performed using Time 1 data. Three of these predictors varied from the first to second time point; viz., clinical cohort, global cognition, and age. Regarding clinical cohort, we observed the expected full spectrum of cognitive status transitions over the longitudinal interval. Accordingly, the initial aMCI cohort includes persons who are in a prodromal stage of AD, but the timing of dementia conversion is not known at baseline. Previous epidemiological research has examined cognitive impairment as a fixed (or time-invariant) predictor of unidimensional frailty trajectories and transitions (i.e., without regard to the timing of clinical progression along an AD spectrum).^11,14^ We contribute to and extend this prior work by characterizing aMCI as an important and differential predictor of latent subgroup classifications and transitions. Follow-up studies could evaluate clinical cohort, global cognition, and age as time-varying predictors (e.g., explore whether patterns of subgroup transitions vary across persons with aMCI who convert to AD versus revert to cognitively unimpaired status).

Second, we assembled data for all UDS visits conducted between January 2005 to March 2020. A potential limitation is that, for this time frame, the NACC database did not contain any participant information that pertained to either the (a) exact age at AD diagnosis or (b) precise diagnosis (autosomal dominant or sporadic (especially LOAD)). As noted above, we restricted our sample to participants aged ≥ 53 years. This approach maximized (a) the number of AD cases, (b) the probability that most, if not all, would represent sporadic cases, and (c) the representativeness of AD cases across the extensive age range represented in our study sample. The percentage of the present sample aged 53–60 years was small (8%), with the remaining participants ranging up to 100 years old. As a result, we expect that LOAD cases are robustly predominant in our AD sample, as would be the case for an AD study spanning the 53–100-year age range. We are not aware of evidence that would suggest the phenomena we are investigating (changes in multimorbidity and deficit burden subgroups over time in aMCI and AD) would be affected by the presence of one versus the other etiological type of AD. However, we note that follow-up research with databases containing large numbers of both earlier and later onset participants could be interesting but would also encounter age-related challenges. Third, we note that prediction analyses were performed by classifying participants into their most likely subgroup at each time point. This approach has demonstrated utility for modeling the developmental course of complex constructs^57,59,97,98^ and can be readily applied in LTA models characterized by large sample sizes and high classification accuracy (i.e., entropy > 0.80).^58,99^ The current study meets these criteria. We note, however, that a potential limitation of this approach is that does not account for all uncertainty (or potential sources of error) in latent subgroup classifications.

### Conclusions

This study is the first to our awareness to distinguish and subsequently track heterogeneity in the developmental course of latent subgroups reflecting multimorbidity and deficit burden within an aMCI and AD cohort. Across time points, we detected a Low Deficit Burden and Moderate Deficit Burden subgroup. At the 2-year follow-up, these subgroups had differentiated into an additional two subgroups that were interpreted as Mild Deficit Burden and Severe Deficit Burden. The domains primarily driving the differentiation of latent subgroups were physical function and instrumental health. Transition analyses revealed that persons initially classified into the Low Deficit Burden subgroup had a high probability of stability, suggesting that persons living with aMCI or AD may not inevitably or quickly present with classifiable and persistent global frailty. Notably, persons initially classified into the Moderate Deficit Burden subgroup evinced multidirectional transition patterns, including stability, progression, and even reversion. The latter results suggest that reductions in multimorbidity and deficit burdens is possible in clinical cohorts living with aMCI or AD. Future research could explore whether this process becomes increasingly challenging once the disabling cascade of frailty is fully established^17^ (e.g., through tracking heterogeneity in Severe Deficit Burden subgroup transitions). Follow-up work could also profitably be directed towards evaluating whether targeting modifiable facets of physical function (e.g., through exercise- or nutrition-based interventions)^67,100^ may have positive downstream effects on attenuating or slowing the rate of multi-domain deficit accumulation. If such deficit reductions are possible, the vision of delaying the incidence of global frailty and related adverse outcomes, even in persons living with cognitive impairment, may be advanced.^72^

## Supplemental Material

sj-docx-1-alz-10.1177_13872877251319547 - Supplemental material for Frailty in motion: Amnestic mild cognitive impairment and Alzheimer's disease cohorts display heterogeneity in multimorbidity classification and longitudinal transitions Supplemental material, sj-docx-1-alz-10.1177_13872877251319547 for Frailty in motion: Amnestic mild cognitive impairment and Alzheimer's disease cohorts display heterogeneity in multimorbidity classification and longitudinal transitions by Linzy Bohn, Yao Zheng, G Peggy McFall, Melissa K Andrew and Roger A Dixon in Journal of Alzheimer's Disease
