# Supplementary material for: Frailty in motion: Amnestic mild cognitive impairment and Alzheimer's disease cohorts display heterogeneity in multimorbidity classification and longitudinal transitions
Source: J Alzheimers Dis. 2025 Mar 2;104(3):732–50. doi: 10.1177/13872877251319547 (PMC12231922; doi:10.1177/13872877251319547)
Supplement: sj-docx-1-alz-10.1177_13872877251319547 - Supplemental material for Frailty in motion: Amnestic mild cognitive impairment and Alzheimer's disease cohorts display heterogeneity in multimorbidity classification and longitudinal transitions [file sj-docx-1-alz-10.1177_13872877251319547.docx]

**Supplemental Material**

**Frailty in motion: Amnestic mild cognitive impairment and Alzheimer’s disease cohorts display heterogeneity in multimorbidity classification and longitudinal transitions**

**Supplemental Methods**

*RG1a: Detecting salient domains of deficit using exploratory and confirmatory factor analyses*

Model fit in the confirmatory factor analysis was determined using the following standard indices: (a) chi-square, for which a good fit would produce a non-significant result (i.e., *p*  > 0.05; indicates that the data do not significantly differ from model-based estimates); (b) the comparative fit index, for which fit is judged by a value of ≥ 0.95 as good and ≥ 0.90 as adequate; (c) root mean square error of approximation, for which fit is judged by a value of ≤ 0.05 as good and ≤ 0.08 as adequate, and (d) Tucker-Lewis Index, for which fit is judged by a value of ≥ 0.95 as good and ≥ 0.90 as adequate.^1^

*RG1b: Applying latent transition analysis to the exploratory factor analysis derived domains*

*Phase 1: Separate latent profile analysis at each time point.* The best-fitting model at each time point was determined by considering interpretability of the results, together with the following model parameters, tests, and fit indices: (a) -2 log-likelihood value (*LL*); (b) number of parameters estimated; (c) Bayesian Information Criterion (BIC); (d) sample-size adjusted BIC (SABIC); Akaike Information Criterion (AIC); (f) adjusted Lo-Mendell-Rubin likelihood ratio test (LMR-LRT); and (g) adjusted Vuong-Lo-Mendell-Rubin likelihood ratio test (VLMR-LRT). ﻿Lower values of BIC, SABIC, and AIC denote better fit. The LMR-LRT and VLMR-LRT test the current model (*k*) against the model with one less latent subgroup (*k*-1) and are interpreted such that a non-significant *p-*value supports selecting the *k*-1 model.^2^ ﻿

**References**

1. Little TD. *Longitudinal structural equation modeling*. New York: NY: Guilford Press, 2013.

2. Nylund-Gibson K and Choi AY. Ten frequently asked questions about latent class analysis. *Transl Issues Psychol Sci* 2018; 4: 440–461.

3. Cummings JL. The Neuropsychiatric Inventory: Assessing psychopathology in dementia patients. *Neurology* 1997; 48: S10-S16.

4. Yesavage JA and Sheikh JI. Geriatric Depression Scale (GDS): Recent evidence and development of a shorter version. *Clin Gerontol* 1986; 5: 165–173.

5. Nasreddine ZS, Phillips NA, Bédirian V, et al. The Montreal Cognitive Assessment, MoCA: A brief screening tool for mild cognitive impairment. *J Am Geriatr Soc* 2005; 53: 695–699.

6. Folstein MF, Folstein SE and McHugh PR. “Mini-mental state”. A practical method for grading the cognitive state of patients for the clinician. *J Psychiatr Res* 1975; 12: 189–198.

7. Monsell SE, Dodge HH, Zhou XH, et al. Results from the NACC Uniform Data Set neuropsychological battery crosswalk study. *Alzheimer Dis Assoc Disord* 2016; 30: 134-139.

8. Gordon EH, Reid N, Khetani IS, et al. How frail is frail? A systematic scoping review and synthesis of high impact studies. *BMC Geriatr* 2021; 21: 719.

9. Morris JC. The Clinical Dementia Rating (CDR): Current version and scoring rules. *Neurology* 1993; 43: 2412–2414.

**Supplemental Table 1.** List of 43 Multimorbidity and Deficit Items Submitted to Exploratory Factor Analysis and used to Calculate an Independent 43-Item Frailty Index

|  | Item | Coding | | |
| --- | --- | --- | --- | --- |
| SR or CE | Stroke | 0 = no; 1 = yes | | |
|  | Diabetes |  | | |
|  | Hypertension |  | | |
|  | Hypercholesterolemia |  | | |
|  | Urinary incontinence |  | | |
|  | Bowel incontinence |  | | |
| SR | Number of medications | 0 = 0-3; 0.5 = 4-7; 1 = 8+ | | |
| CE | Difficulty writing checks, paying bills, or balancing a check book | 0 = normal, N/A; 0.33 = difficulty, but independent; 0.66 = requires assistance; 1 = dependent ^a^ | | |
|  | Difficulty assembling tax records, business affairs, or other papers |  |  |  |
|  | Difficulty shopping alone for clothes, household necessities, or groceries |  |  |  |
|  | Difficulty playing a game of skill |  | | |
|  | Difficulty heating water, making a cup of coffee, turning off the stove |  | | |
|  | Difficulty preparing a balanced meal |  | | |
|  | Difficulty keeping track of current events |  | | |
|  | Difficulty paying attention to/understanding TV program, book, magazine |  | | |
|  | Difficulty remembering appointments, family occasions, holidays, medications | |  | |
|  | Difficulty traveling out of the neighborhood, driving, or arranging to take public transportation | | |  |
| CE | Walking changed not due to injury or arthritis | 0 = no; 1 = yes | | |
|  | Falls more than usual |  | | |
|  | Tremor |  | | |
|  | Slowing of motor movements |  | | |
| CE | Engages in repetitive activities (pacing, handling buttons, wrapping string) | 0 = no, N/A; 1 = yes ^b^ | | |
|  | Nighttime behaviors: awakens in the night, rises too early, excessive naps |  |  |  |
|  | Appetite: changes in weight or food preferences |  |  |  |
|  | Delusions |  | | |
|  | Depression and/or dysphoria |  | | |
|  | Anxiety |  | | |
|  | Agitation and/or aggression |  | | |
|  | Apathy and/or indifference |  | | |
|  | Irritability and/or lability |  | | |
|  | Disinhibition |  | | |
| M | Body mass index (kg/m^2^) | 0 = 18.5-25; 0.5 = 25.1 to < 30;  1 = < 18.5 or ≥ 30 | | |
| SR | Dropped many activities and interests | 0 = no; 1 = yes ^c^ | | |
|  | Feel that life is empty |  | | |
|  | Often get bored |  | | |
|  | Afraid something bad is going to happen to you |  | | |
|  | Often feel helpless |  | | |
|  | Prefer to stay home rather than going out and doing new things |  | | |
|  | Feel worthless |  | | |
|  | Feel that your situation is hopeless |  | | |
|  | Feel that most people are better off than you |  | | |
| SR | Feel full of energy | 0 = yes; 1 = no ^c^ | | |
|  | Basically satisfied with life |  | | |

^a^ Reported as change over the past four weeks; ^b^ Item from the Neuropsychiatric Inventory Questionnaire.^3^ ^c^ Item from the Geriatric Depression Scale.^4^ SR: self-reported; CE: clinician evaluated; M: measured.

**Supplemental Table 2.** Model Fit Indices for the Confirmatory Factor Analysis Conducted at Each Time Point

| 5-Factor Model | ﻿χ^2^ | *df* | *p* | RMSEA | CFI | TLI |
| --- | --- | --- | --- | --- | --- | --- |
| **Random subset of overall study sample (50%)** | | | |  |  |  |
| Time 1 | 1213.81 | 485 | <0.001 | 0.03 (0.03 - 0.03) | 0.89 | 0.88 |
| Time 2 | 1337.80 | 485 | <0.001 | 0.03 (0.03 - 0.04) | 0.88 | 0.87 |
| **Entire study sample** |  |  |  |  |  |  |
| Time 1 | 1794.70 | 485 | <0.001 | 0.03 (0.03 - 0.03) | 0.90 | 0.89 |
| Time 2 | 2138.87 | 485 | <0.001 | 0.03 (0.03 - 0.04) | 0.87 | 0.86 |

RMSEA is shown with 90% confidence intervals. Results verified that a solution comprised of the five exploratory factor analysis derived domains provided adequate-to-good fit to the data at each time point (see Supplemental Figures 3 and 4 for model depiction). χ^2^, chi-square test of model fit; *df*, degrees of freedom for model fit; RMSEA, root mean square error of approximation; CFI, comparative fit index; TLI, Tucker Lewis Index.

**Supplemental Table 3.** Model Fit Indices for Latent Profile Solutions at Each Time Point

| *n* subgroups | -2*LL* | npar | AIC | BIC | SABIC | LMR | VLMR | Entropy |
| --- | --- | --- | --- | --- | --- | --- | --- | --- |
| **Model: Time 1** | | |  |  |  |  |  |  |
| 1 | -2,862.42 | 20 | -2822.42 | -2701.81 | -2765.35 | -- | -- | -- |
| **2** | **-4,558.66** | **26** | **-4506.66** | **-4349.86** | **-4432.48** | **<0.001** | **<0.001** | **0.99** |
| 3 | -6,297.75 | 32 | -6,297.75 | -6233.75 | -6040.77 | 0.11 | 0.11 | 1.0 |
| **Model: Time 2** | | |  |  |  |  |  |  |
| 1 | 610.98 | 20 | 650.98 | 771.60 | 708.05 | -- | -- | -- |
| 2 | -511.24 | 26 | -459.24 | -302.44 | -385.05 | <0.001 | <0.001 | 0.91 |
| 3 | -1,260.28 | 32 | -1196.28 | -1003.30 | -1104.97 | <0.001 | <0.001 | 0.97 |
| **4** | **-1,640.11** | **38** | **-1564.11** | **-1334.94** | **-1455.68** | **<0.001** | **<0.001** | **0.91** |
| 5 | -2,138.66 | 44 | -2050.66 | -1,785.31 | -1925.117 | 1.0 | 1.0 | 0.92 |

The best-fitting model is bolded. -2﻿*LL*, -2 log-likelihood; npar, number of parameters free; AIC, Akaike information criterion; BIC, Bayesian information criterion; SABIC, sample size adjusted BIC; LMR, adjusted Lo-Mendell-Rubin likelihood ratio test; VLMR, adjusted Vuong-Lo-Mendell-Rubin likelihood ratio test.

**Supplemental Table 4.** Model Fit Indices for Longitudinal Measurement Invariance Tests

|  | -2*LL* | npar | AIC | BIC | SABIC | Δ -2*LL* | Δ AIC | Δ BIC |
| --- | --- | --- | --- | --- | --- | --- | --- | --- |
| Model 1^a^ | -6198.77 | 64 | -6070.77 | -5684.80 | -5888.16 | -- | -- | -- |
| **Model 2^b^** | **-6635.85** | **54** | **-6527.85** | **-6202.19** | **-6373.77** | **-437.08** | **-517.39** | **-457.08** |
| Model 3^c^ | -5733.36 | 39 | -5655.36 | -5420.16 | -5544.08 | 902.49 | 782.03 | 872.50 |

The best-fitting model is bolded. ^a^ Within-subgroup indicator means for the five exploratory factory analysis (EFA) derived domains were free to vary at Time 2. ^b^ Structurally invariant model: Within-subgroup indicator means for the five EFA-derived domains were constrained to be equal to the corresponding domain at Time 2. ^c^ Dispersion invariance model: Within-subgroup indicator means and variances for the five EFA-derived domains were constrained to equality at Time 2. -2 ﻿*LL*, -2 log-likelihood; npar, number of parameters free; AIC, Akaike information criterion; BIC, Bayesian information criterion; SABIC, sample size adjusted BIC; Δ, change in the associated parameter.

**Supplemental Table 5.** Standardized Mean Differences Across Latent Subgroups in the Five Exploratory Factor Analysis Derived Domains

|  | Latent Subgroup Comparison | | | | | |
| --- | --- | --- | --- | --- | --- | --- |
| EFA-Derived Domain | SEV–MOD | SEV–MILD | SEV–LOW | MOD–MILD | MOD–LOW | MILD–LOW |
| Cardiovascular symptoms | 0.05 | 0.09 | 0.43 | 0.05 | 0.43 | 0.38 |
| Instrumental health | 0.70 | 0.97 | **1.47** | 0.20 | 0.89 | 0.69 |
| Behavioral disturbances | 0.36 | 0.54 | 0.74 | 0.20 | 0.44 | 0.24 |
| Emotional well-being | 0.15 | 0.37 | 0.43 | 0.24 | 0.31 | 0.09 |
| Physical function | **8.98** | **15.20** | **18.70** | **6.96** | **11.27** | **4.63** |

Model estimated indicator means for the Low Deficit Burden and Moderate Deficit Burden subgroups were constrained to equality over time (for details see Supplemental Table 4). Standardized mean differences > 2.0 indicate a less than 20% overlap in subgroup-specific distributions and a high degree of separation on the associated EFA-derived domain, whereas values < 0.85 indicate more than 50% overlap and a low degree of separation on the associated EFA-derived domain. Bolded values represent indicators with a moderate-to-high degree of separation. Indicators are coded such that higher values denote greater multimorbidity and deficit burden in the corresponding EFA-derived domain. SEV, Severe Deficit Burden; MOD, Moderate Deficit Burden; MILD, Mild Deficit Burden; Low, Low Deficit Burden.

**Supplemental Table 6.** Baseline Clinical and Demographic Characteristics Disaggregated by Latent Subgroups

|  | Time 1 Latent Subgroup | | Time 2 Latent Subgroup | | | |
| --- | --- | --- | --- | --- | --- | --- |
| Characteristic | LOW | MOD | LOW | MILD | MOD | SEV |
| *n*(%) | 2,790 (91%) | 284 (9%) | 1,714 (56%) | 654 (21%) | 571 (19%) | 135 (4%) |
| Inter-wave interval (days) | 779.81 (78.71) | 782.01 (80.65) | 778.68 (77.36) ^c^ | 776.41 (74.95) ^c^ | 782.32 (84.16) ^c^ | 804.56 (89.19) ^d, **^ |
| *n*(%) self-reported female | 4,466 (53%) | 137 (48%) | 876 (51%) ^c^ | 375 (57%) ^d^ | 283 (50%) ^c^ | 69 (51%) ^c,d^ ^**^ |
| Age (y) | 74.23 (8.63) | 79.25 (7.98) ^***^ | 72.72 (8.49) ^c^ | 76.31 (8.36) ^d^ | 77.96 (8.22) ^d^ | 78.19 (7.90) ^d, ***^ |
| Education (y) | 15.12 (3.36) | 14.64 (3.43) ^*^ | 15.27 (3.22) ^c^ | 14.77 (3.55) ^d,e^ | 15.07 (3.53) ^c,d^ | 14.11 (3.36) ^e, ***^ |
| *n*(%) non-Hispanic White | 2,531 (91%) | 251 (88%) | 1,564 (91%) | 593 (91%) | 508 (89%) | 117 (87%) |
| *n*(%) married | 2,021 (72%) | 194 (68%) | 1,292 (76%) ^c^ | 449 (69%) ^d^ | 386 (68%) ^d^ | 88 (65%) ^d, ***^ |
| *n*(%) in private residence | 2,552 (92%) | 243 (86%) ^***^ | 1,584 (94%) ^c^ | 588 (92%) ^c,d^ | 505 (90%) ^d^ | 118 (89%) ^d, **^ |
| MMSE^~^ | 23.42 (4.64) | 21.94 (5.54) ^***^ | 24.00 (4.39) ^c^ | 23.25 (4.46) ^d^ | 21.97 (5.18) ^e^ | 19.92 (5.87) ^f, ***^ |
| CDR^®^ | 0.77 (0.40) | 1.02 (0.54) ^***^ | 0.71 (0.34) ^c^ | 0.80 (0.39) ^d^ | 0.95 (0.50) ^e^ | 1.09 (0.54) ^f, ***^ |
| *n*(%) *APOE* ﻿ε4 carriers ^^^ | 1,419 (55%) | 117 (49%) ^**^ | 912 (61%) ^c^ | 323 (57%) ^c^ | 234 (49%) ^d^ | 67 (60%) ^c, ***^ |
| 43-item frailty index ^^^^ | 0.22 (0.12) | 0.37 (0.12) ^***^ | 0.26 (0.13) ^^, c^ | 0.33 (0.13) ^d^ | 0.42 (0.13) ^e^ | 0.55 (0.14) ^f, ***^ |

Results presented as mean (standard deviation) or *n*(%) of the sample with the associated characteristic. *p-*values are based on independent sample *t*-tests, one-way ANOVA, or chi-square tests, as appropriate. ﻿We adjusted for multiple comparisons using post-hoc Tukey tests or Games-Howell tests, as appropriate.  ^~^A subset of each sample was administered the Montreal Cognitive Assessment (MoCA)^5^ in lieu of the Mini-Mental State Exam (MMSE);^6^ education-adjusted scores on the MoCA were converted to an equivalent continuous MMSE score using published conversion tables derived from the National Alzheimer’s Coordinating Center Uniform Data Set.^7^ ^^^ Results are based on 2,649 participants who were genotyped. ^^^^ Values for the 43-item frailty index are reported for background characterization purposes only; higher values represent increasing levels of global frailty (values > 0.21 can be used to assign frailty status).^8^ ^c,d,e,f^ ﻿Values with different superscripts differ significantly from one another. LOW: Low Deficit Burden; MILD: Mild Deficit Burden; MOD: Moderate Deficit Burden; SEV: Severe Deficit Burden; Sig: significance; aMCI: amnestic mild cognitive impairment; AD: Alzheimer’s disease; CDR®: Clinical Dementia Rating Scale;^9^ ﻿*APOE*: Apolipoprotein E. ^***^*p* < 0.001; ^**^*p* < 0.01; ^*^*p* < 0.05

**Supplemental Table 7.** Patterns of Latent Subgroup Transitions Disaggregated by the two Baseline Clinical Cohorts: Amnestic Mild Cognitive Impairment and Alzheimer’s Disease

| **Subgroup transition pattern** | **aMCI (*n* = 878)** | **AD (*n* = 2,196)** | ***p*** |
| --- | --- | --- | --- |
| Subgroup Stability (*n* = 1,848) | 624 (71%) | 1,224 (55%) | < 0.05 |
| Subgroup Progression (*n* = 1,156) | 240 (27%) | 916 (42%) | < 0.05 |
| Subgroup Reversion (*n* = 70) | 14 (2%) | 1. (3%) | > 0.05 |

In the Table, we collapsed across the two Time 1 subgroups (Low Deficit Burden, Moderate Deficit Burden) and separately display the proportion of participants within each of the baseline clinical cohorts (amnestic mild cognitive impairment (aMCI), Alzheimer’s disease (AD)) that evinced stability (i.e., remained in the same subgroup at follow-up), progressed (i.e., transitioned to a subgroup characterized by higher multimorbidity and deficit burdens), or reverted (i.e., to a “healthier” subgroup characterized by lower multimorbidity and deficit burdens). We compared these proportions using a chi-square test (χ^2^ = 61.52, *p* < 0.001).


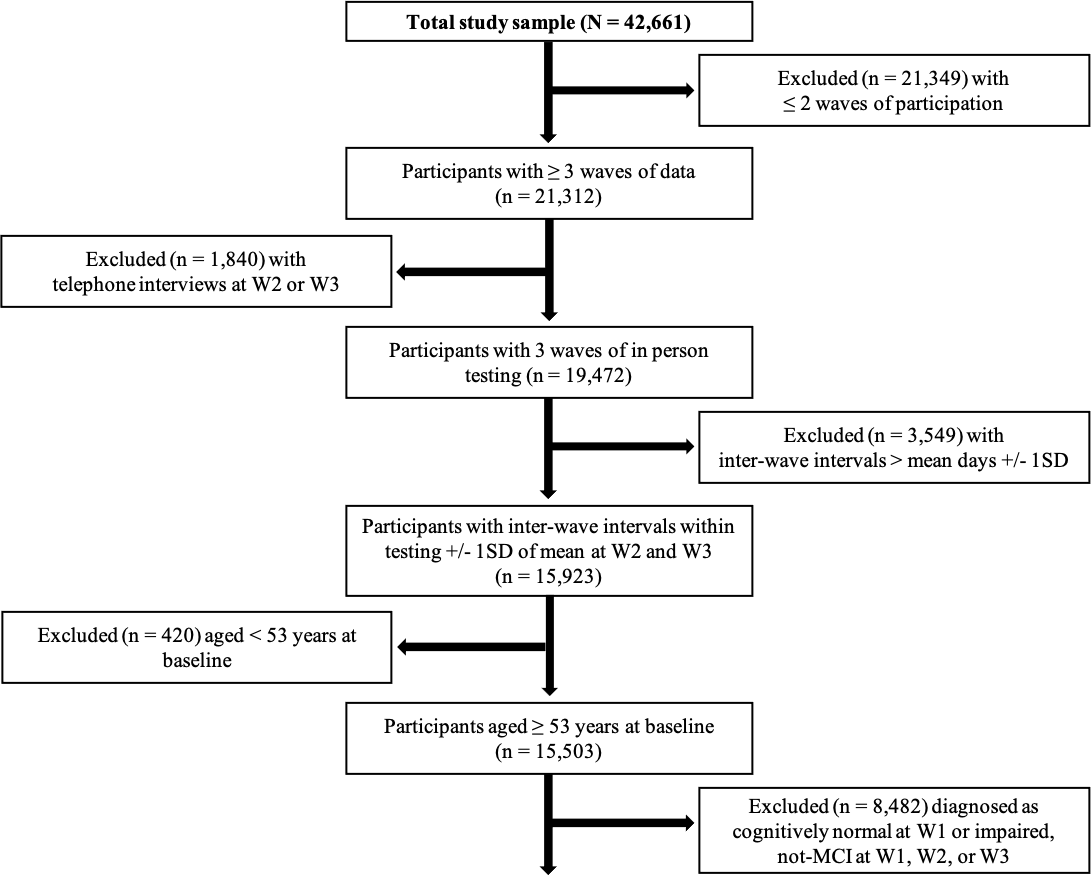

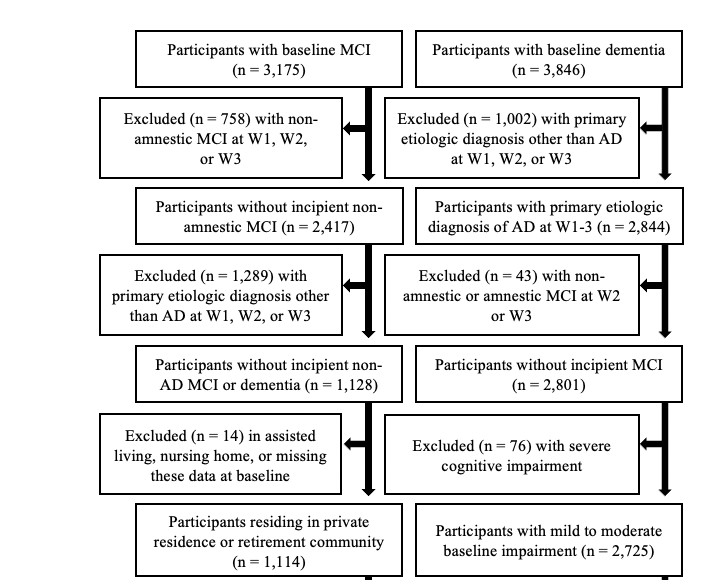


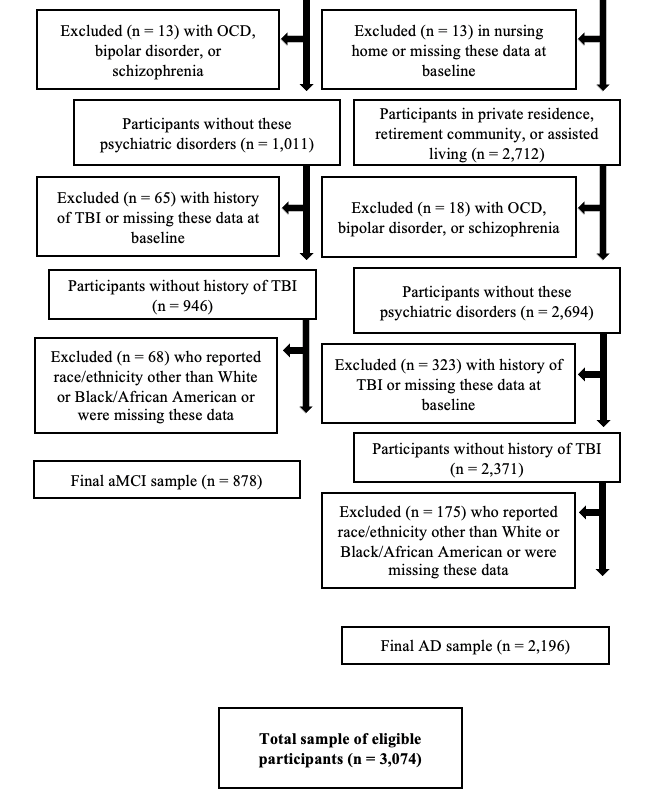


**Supplemental Figure 1.** Flow diagram of study participants.

**
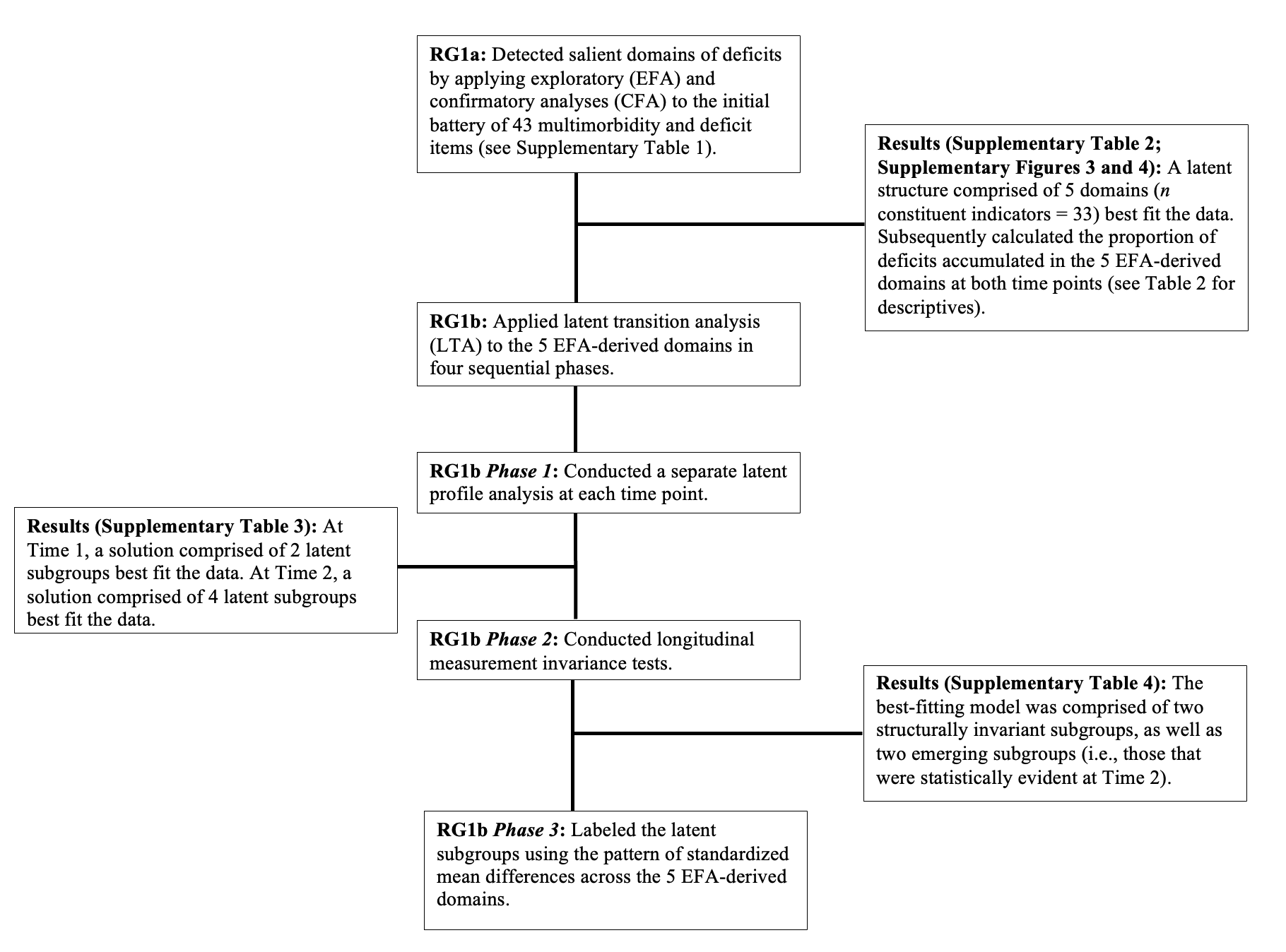

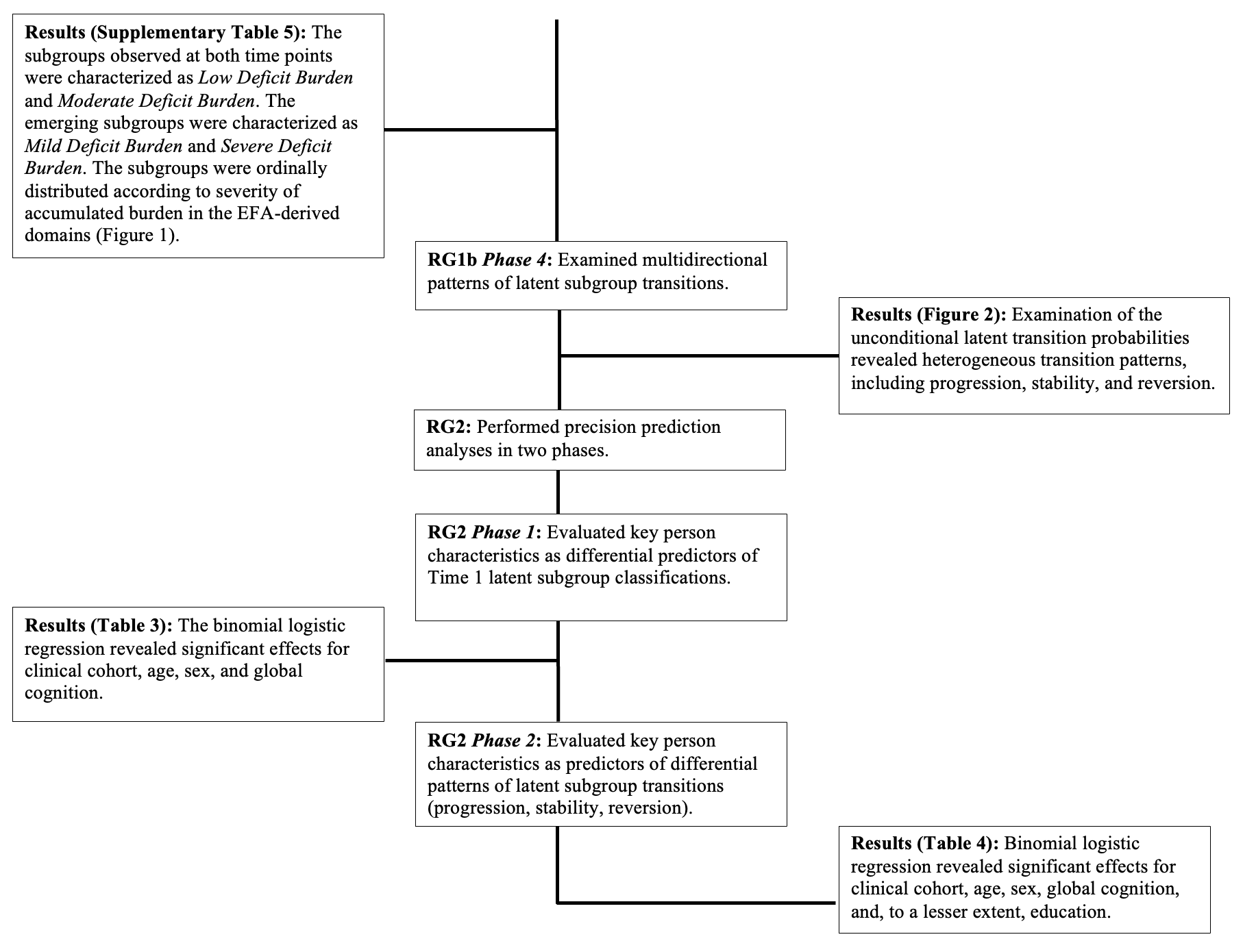
**

**Supplemental Figure 2.** Analytic workflow of the two research goals (RG).


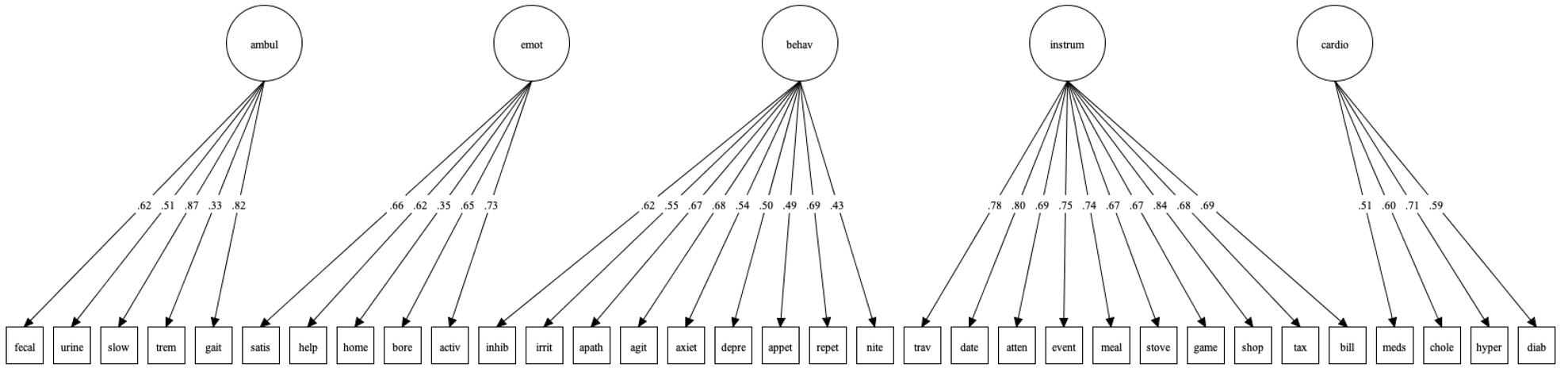


phys

**Supplemental Figure 3.** Time 1 confirmatory factor analysis model conducted with the entire study sample (*n* of retained domains (or factors) = 5; total *n* of constituent indicators = 33). Abbreviations: Phys, physical function; Emot, emotional well-being; Behav, behavioral disturbances; Instrum, instrumental health; Cardio, cardiovascular symptoms; Fecal, bowel incontinence; Urine, urinary incontinence; Slow, slowing of motor movements; Trem, tremor; Gait, walking changed; Satis, satisfied with life; Help, feel helpless; Home, prefer to stay home; Bore, often bored; Activ, dropped many activities; Inhib, disinhibition; Irrit, irritability; Apath, apathy; Agit, agitation; Anxiet; anxiety; Depre, depression; Appet; changes in appetite; Repet, repetitive activities; Nite, nighttime behaviors; Trav, difficulty traveling; Date, difficulty remembering appointments; Atten, difficulty paying attention; Event, difficultly tracking current events; Meal, difficulty preparing meal; Stove, difficulty turning off stove; Game, difficulty playing games; Shop, difficulty shopping alone; Tax, difficulty assembling tax records; Bill, difficulty paying bills; Meds, total number of medications; Choles, hypercholesterolemia; Hyper, hypertension; Diab, diabetes. Standardized factor loadings are shown. All loadings were significant at *p* < 0.05. Latent covariances and residuals are not depicted. Response scales for the constituent indicators are reported in Supplemental Table 1.


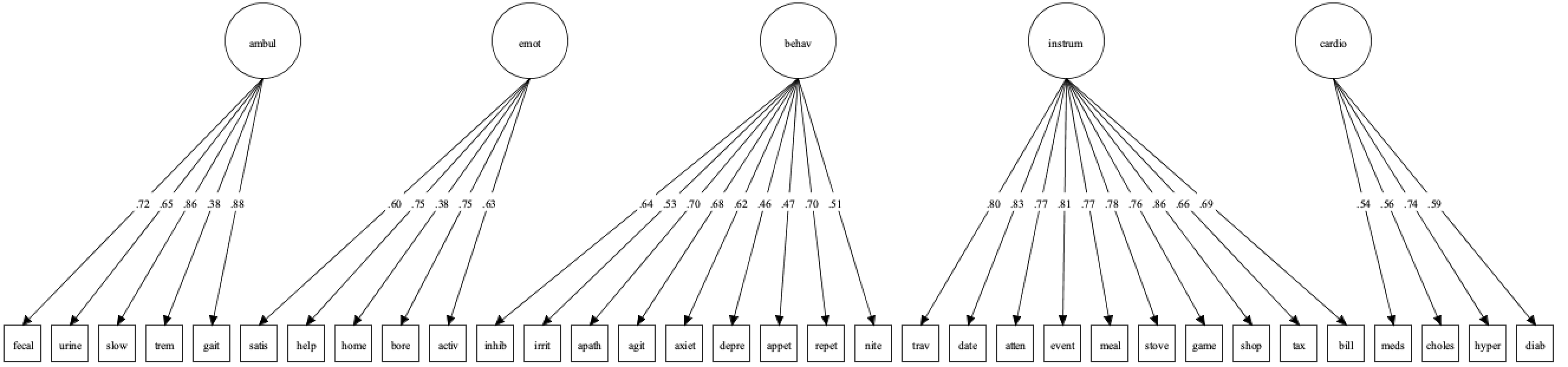


phys

**Supplemental Figure 4.** Time 2 confirmatory factor analysis model conducted with the entire study (*n* of retained domains (or factors) = 5; total *n* of constituent indicators = 33). Abbreviations: Phys, physical function; Emot, emotional well-being; Behav, behavioral disturbances; Instrum, instrumental health; Cardio, cardiovascular symptoms; Fecal, bowel incontinence; Urine, urinary incontinence; Slow, slowing of motor movements; Trem, tremor; Gait, walking changed; Satis, satisfied with life; Help, feel helpless; Home, prefer to stay home; Bore, often bored; Activ, dropped many activities; Inhib, disinhibition; Irrit, irritability; Apath, apathy; Agit, agitation; Anxiet; anxiety; Depre, depression; Appet; changes in appetite; Repet, repetitive activities; Nite, nighttime behaviors; Trav, difficulty traveling; Date, difficulty remembering appointments; Atten, difficulty paying attention; Event, difficultly tracking current events; Meal, difficulty preparing meal; Stove, difficulty turning off stove; Game, difficulty playing games; Shop, difficulty shopping alone; Tax, difficulty assembling tax records; Bill, difficulty paying bills; Meds, total number of medications; Choles, hypercholesterolemia; Hyper, hypertension; Diab, diabetes. Standardized factor loadings are shown. All loadings were significant at *p* < 0.05. Latent covariances and residuals are not depicted. Response scales for the constituent indicators are reported in Supplemental Table 1.
